# Interplay Between Stereochemically Active Lone Pair Repulsions, Sigma Hole Interactions, and Delocalized Redox Processes in Topochemical Fluoride‐Ion Insertion

**DOI:** 10.1002/anie.202507650

**Published:** 2025-06-23

**Authors:** Anindya Pakhira, Shruti Hariyani, George Agbeworvi, Jaime R. Ayala, Conan Weiland, Cherno Jaye, Daniel A. Fischer, Lu Ma, Sarbajit Banerjee

**Affiliations:** ^1^ Department of Chemistry Texas A&M University College Station TX 77843 USA; ^2^ Material Measurement Laboratory National Institute of Standards and Technology Gaithersburg MD 20899 USA; ^3^ National Synchrotron Light Source II Brookhaven National Laboratory Upton NY 11973 USA; ^4^ Laboratory for Inorganic Chemistry, Department of Chemistry and Applied Biosciences ETH Zurich Vladimir‐Prelog‐Weg 2 Zürich CH‐8093 Switzerland; ^5^ Laboratory for Battery Science, PSI Center for Energy and Environmental Sciences Paul Scherrer Institute Forschungsstrasse 111 Villigen PSI CH‐5232 Switzerland

**Keywords:** Anion insertion, Dative interactions, Energy storage, Fluoride‐ion batteries, Sigma‐hole interactions, Unconventional redox

## Abstract

Topochemical insertion/extraction of cations has emerged as a generalizable strategy for modulating the crystal and electronic structure of periodic solids. In contrast, strategies for topochemical anion insertion are poorly explored and fundamental principles for designing insertion hosts to accommodate anions remain scarce. Here, we observe reversible room‐temperature fluoride‐ion insertion within tunnels of Sn_2_TiO_4_ defined by the stereochemical expression of Sn 5*s* ^2^ lone pairs. X‐ray scattering studies of fluoride‐ion‐insertion‐induced crystal structure modulation and X‐ray absorption/emission spectroscopy probes of electronic structure along with magnetic susceptibility measurements and first‐principles calculations are used to decipher design principles underpinning reversible fluoride‐ion insertion and bulk diffusion. Fluoride‐ion insertion is enabled by a combination of a large, polarizable tunnel, delocalized redox at Ti─O─Sn centers, inherent repulsion between the fluoride‐ion and Sn 5*s* ^2^ electron lone pairs, and the formation of dative interactions between Sn‐centered σ‐holes and fluoride‐ions, yielding a reversible capacity of 0.5 fluoride‐ions per Sn_2_TiO_4_ formula unit. Our results demonstrate that the complex interplay between dative interactions and stereochemically active lone pair repulsions is critical to defining the thermodynamics and kinetics controlling fluoride‐ion insertion and diffusion. As such, the design of fluoride‐ion insertion hosts for anion batteries requires site‐selective modification to modulate lattice—ion interactions.

## Graphical abstract

Fluoride‐ion insertion and diffusion within Sn_2_TiO_4_ is mediated by the complex interplay between the formation of Sn─F dative interactions and redox processes delocalized across Ti─O─Sn hybrid states counterbalanced by repulsions between fluoride‐ions and stereochemically expressed Sn 5*s* ^2^ electron lone pairs. The coupled ion—electron transfer and transport processes are mediated along large tunnels that themselves result from stereochemical expression and repulsions between Sn 5*s* ^2^ electron lone pairs.

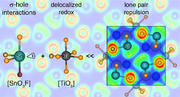

## Introduction

Topochemical ion insertion, specifically of alkali metal ions, into periodic solids underpins much of modern electrochemical energy storage and involves accommodation of guest ions in interstitial sites whilst preserving the overall atomic connectivity of the crystal lattice. Charge balance is achieved through closely coupled ion and electron transport processes.^[^ ^1^, ^2^ ^]^ As compared to conversion chemistry alternatives that potentially afford higher specific energy capacities,^[^ ^3^, ^4^ ^]^ insertion chemistries require less bond‐breaking and forming, engender lower stress accumulation as a result of more modest volume changes, encumber lower voltage hysteresis, and generally exhibit greater reversibility and faster kinetics of ion and electron transport.^[^ ^5^, ^6^ ^]^ In contrast, the insertion chemistry of *anions* is much less explored^[^ ^7^, ^8^, ^9^, ^10^, ^11^ ^]^ and scarce little has been derived in terms of generalizable design principles. In analogy with Li‐ion insertion, the discovery and design of fluoride‐ion insertion hosts could potentially enable electrochemical energy storage using earth‐abundant fluoride‐ions as charge carriers and enable exploration of battery chemistries that entirely circumvent metal electrodeposition/plating.^[^ ^12^, ^13^, ^14^, ^15^ ^]^

Fluoride‐ion conversion batteries, which rely on a conversion reaction between a metal and a metal fluoride as per *M* + *x*F^−^ ↔ *M*F*_x_ *+* x*e^−^, represent an intriguing energy storage concept.^[^ ^15^ ^]^ Such constructs boast exceptionally high theoretical energy densities as a result of their ability to accommodate more than one mole of charge carrier by conversion of a metal to a bi‐ or trifluoride. However, the significant volume change (Δ*V*) experienced by the metal upon conversion to the fluoride, as high as 311% for Fe ↔ FeF_3_, represents a major drawback.^[^ ^16^ ^]^ This repeated expansion/contraction causes the electrode to pulverize, which leads to problems in reversibility and cyclability, and eventually induces battery failure.^[^ ^16^, ^17^, ^18^ ^]^ As such, the development of insertion‐based fluoride‐ion electrodes has emerged as an urgent imperative.^[^ ^19^ ^]^

The ability to topochemically accommodate ions within the vacancies, interstitial positions, or interlayer sites of a host compound without inducing significant changes in symmetry is expected to mitigate the detrimental volume changes experienced by conversion‐type electrodes. Yamamoto and colleagues have recently reported fluoride‐ion insertion accompanied by both cation and anion redox to enable charge compensation, as well as stabilization of molecular dinitrogen, in Cu_3_N, which yields a high reversible capacity of ca. 550 mAh g^−1^.^[^ ^19^ ^]^ However, beyond this most recent example, the development of new fluoride‐ion electrodes has been somewhat limited to certain crystal structure families. The most widely investigated class of fluoride‐ion electrodes crystallize in the Ruddlesden‐Popper K_2_NiF_4_ (*I*4/*mmm*) structure type. For example, both LaSrMnO_4_ and La_2_CoO_4_ have both been reported to accommodate more than one mole of fluoride ions per formula unit, but anion insertion induces a symmetry reduction to *P*4/*nmm* (Δ*V* = 17.3%) and *C*2*/m* (Δ*V* = 13.5%), respectively. Recent progress has also been made by inserting fluoride‐ions in perovskites such as ReO_3_ and CsMnFeF_6_.^[^ ^11^, ^20^ ^]^ Again, the insertion of fluoride‐ions induces a complex structural transformation to lower symmetry structures. While these examples depict outstanding progress, the symmetry reduction and volume changes experienced by these systems induce stress accumulation, resulting in limited utilization of available capacities^[^ ^21^ ^]^ and cycling‐induced degradation.^[^ ^4^ ^]^

The Schafarzikite mineral system, with general formula *MA*_2_O_4_ is distinctive for being able to insert anions within vacant interstitial positions.^[^ ^22^ ^]^ FeSb_2_O_4_ inserts up to one mole of fluoride‐ions upon treatment with XeF_2_ at room temperature with a < 1% change in volume.^[^ ^23^, ^24^ ^]^ One unique feature of the Schafarzikite family is the presence of *ns* ^2^ stereochemically active lone pairs of electrons on the *p*‐block cations. These *ns* ^2^ electrons are vital for opening large one‐dimensional tunnels along the [001] direction that create the interstitial positions for anion insertion. However, it is not well understood how the inserted anion interacts with the *p*‐block cation and how these interactions potentially impact diffusion in solid‐state electrodes. In this work, we seek to derive generalizable design principles for fluoride‐ion insertion hosts based on a detailed mechanistic examination of the topochemical (de)fluoridation of isostructural Schafarzikite Sn_2_TiO_4_. We examine the role of redox processes at Ti sites, which are less than tetravalent as a result of electron density delocalization and are formally oxidized upon fluoride‐ion insertion. Fluoride‐ion insertion and the simultaneous oxidation of Ti has been investigated by hard X‐ray photoemission spectroscopy (HAXPES), extended X‐ray absorption fine structure spectroscopy (EXAFS), and magnetic susceptibility measurements. Elucidation of local atomistic and electronic structure of Sn centers using HAXPES, O K‐edge X‐ray absorption near edge structure (XANES) spectroscopy, and first‐principles calculations revealed the formation of Sn─F dative bonds.

## Results and Discussion

### Synthesis, Structure Modulation, and Structural Implications of Stereochemical Activity

The synthesis of Sn^2+^‐containing Sn_2_TiO_4_ requires careful consideration to avoid the disproportionation of SnO to Sn and SnO_2_.^[^ ^25^ ^]^ Direct solid‐state synthesis from a 1:2 (mol/mol) mixture of anatase TiO_2_ and SnO was unsuccessful and yielded significant SnO_2_ and β‐Sn_0.875_ impurities at 600 °C and a mixture of SnO_2_, β‐Sn_0.875_, and rutile TiO_2_ at 800 °C. (Figure S1). Sn_2_TiO_4_ was not observed in either reaction. While microwave reactions^[^ ^26^ ^]^ have shown some promise in obtaining Sn_2_TiO_4_, we have instead used a topotactic ion‐exchange reaction with Ba_2_TiO_4_ in an effort to obtain a product with micrometer‐sized or sub‐micron dimensions to enable homogeneous fluoride‐ion insertion capabilities.^[^ ^24^, ^27^ ^]^ Ba_2_TiO_4_ was first prepared via solid‐state reaction starting from BaCO_3_ and TiO_2_ as per Equation (1).

(1) $$2{\mathrm{BaCO}}_{3}\left(s\right)+{\mathrm{TiO}}_{2}\left(s\right)\to \;{\mathrm{Ba}}_{2}{\mathrm{TiO}}_{4}\left(s\right)+2{\mathrm{CO}}_{2}\;\left(g\right)$$

The purity of the obtained Ba_2_TiO_4_ was confirmed (Figure S2) prior to combining it with a SnClF peritectic mixture and annealing at 400 °C to obtain Sn_2_TiO_4_ as per Equations (2).^[^ ^28^, ^29^, ^30^ ^]^

(2) $$\begin{array}{ccc} & & {\mathrm{Ba}}_{2}{\mathrm{TiO}}_{4}\left(s\right)+{\mathrm{SnCl}}_{2}\left(s\right)+{\mathrm{SnF}}_{2}\left(s\right)\;\\ & & \;\to {\mathrm{Sn}}_{2}{\mathrm{TiO}}_{4}\left(s\right)+2\mathrm{BaClF}\left(s\right) \end{array}$$

**Figure 1:**
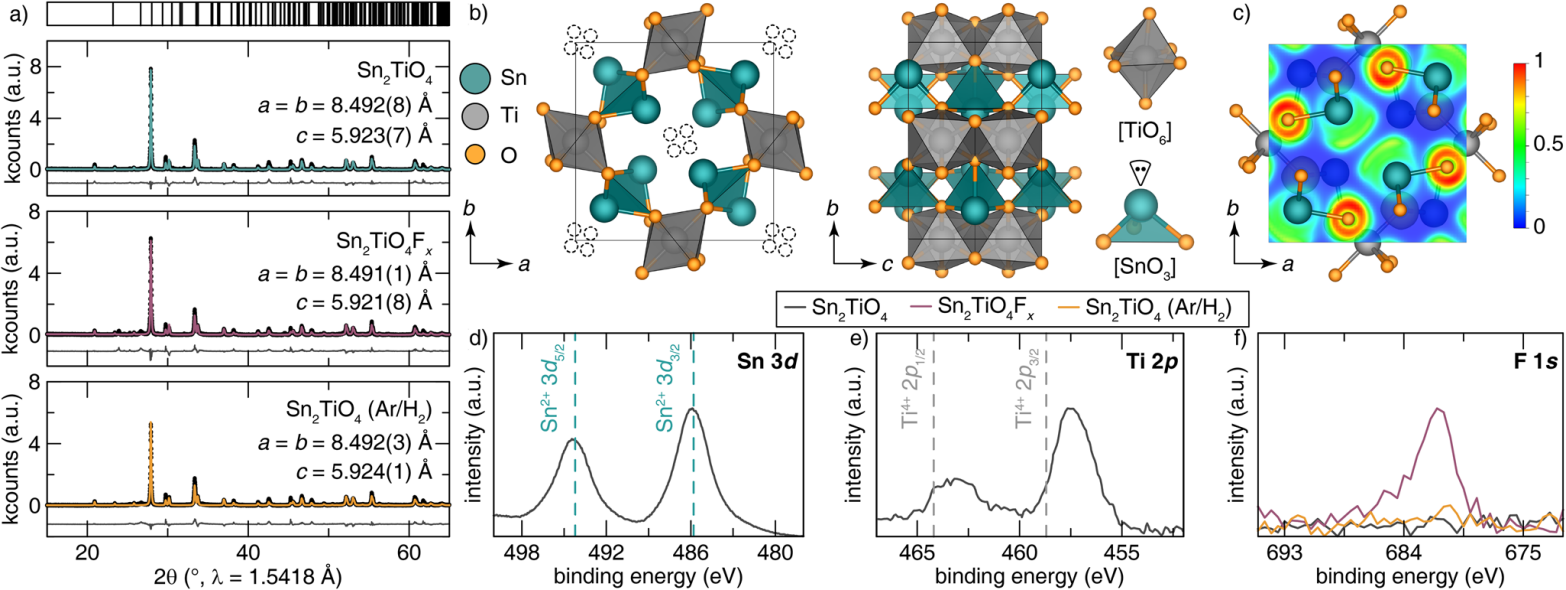
a) The Rietveld refined powder X‐ray diffraction patterns of as‐prepared Sn_2_TiO_4_ (green), Sn_2_TiO_4_F*_x_* (pink), and defluoridated Sn_2_TiO_4_ (orange) indicate that the average structure is preserved across the (de)fluoridation treatments and that the crystal structure reversibly contracts upon fluoride‐ion insertion. b) Crystal structure rendition of Sn_2_TiO_4_ viewed along *c* and *a*. The fluoride‐ion interstitial positions are shown by the dashed black lines. Stereochemically active lone pairs (LPs) on the Sn centers are schematically depicted provided to show the local coordination environment of [SnO_3_LP] pseudo‐tetrahedra where lone pairs occupy one of the vertices. c) Electron localization function representation of Sn_2_TiO_4_ highlighting the stereochemically active lone pairs on Sn sites that define a 1D tunnel along the *c* axis. The energy cutoff for the calculation was 1 × 10^−6^ eV. d) Core level Sn 3*d*_5/2_ and 3*d*_3/2_ HAXPES spectrum of Sn_2_TiO_4_ shows excellent agreement with the positions of the Sn 3*d*_5/2_ and 3*d*_3/2_ peaks from a SnO standard (dashed green line, from ref [31]). e) The Ti 2*p*_3/2_ and 2*p*_1/2_ HAXPES spectrum of Sn_2_TiO_4_ shows that the Ti atoms are not in a tetravalent oxidation state. The HAXPES peak positions of TiO_2_ (from ref [32]) are denoted by dashed grey lines for comparison. f) The F 1s HAXPES spectrum of Sn_2_TiO_4_ (grey), Sn_2_TiO_4_F*_x_* (pink), and defluoridated Sn_2_TiO_4_ (orange) acquired at 2 keV corroborates the successful reversible bulk fluoridation and defluoridation of the title compound.

The phase purity of the resulting product was confirmed using powder X‐ray diffraction (Figure S3). The Rietveld refinement of Sn_2_TiO_4_ is provided in Figure 1a and shows excellent agreement with the published crystal structure (ICSD #163230). The refinement statistics and the refined crystal structure data are provided in Tables S1 and S2, respectively, attesting to stoichiometric replacement of Ba with Sn in the Sn_2_TiO_4_ product.

Sn_2_TiO_4_ crystallizes in the tetragonal *P*4_2_/*mbc* Minium Pb_3_O_4_ structure type.^[^ ^28^ ^]^ The structure, shown in Figure 1b, is built from edge‐sharing, Jahn‐Teller‐distorted [TiO_6_] octahedra, which have refined equatorial and axial Ti─O bond lengths of 2.031(2) and 1.893(4) Å, respectively. The [TiO_6_] octahedra corner‐share with [SnO_3_] pseudo‐tetrahedra where the vertex of the tetrahedra is occupied by the stereochemically active 5*s* ^2^ lone pairs of electrons of Sn^2+^ (vide infra). The stereochemical expression of the 5*s* ^2^ lone pairs of electrons is clearly observed in the electron localization function (ELF) map shown in Figure 1c. These lone pairs of electrons are projected toward each other within the crystal structure; the resulting electron—electron repulsion opens up a large polarizable tunnel along the *c* direction that is approximately 3.705 Å wide. These tunnels host vacant interstitial positions where fluoride‐ions can be accommodated (Figure 1b).

Scanning electron microscopy (SEM) reveals that Sn_2_TiO_4_ has an irregular rod‐like particle morphology and energy‐dispersive X‐ray (EDX) spectroscopy and elemental mapping (Figure S4) suggest the nearly complete removal of the BaCl_2_ and BaF_2_ salts. In addition, the SEM images indicate that micron to sub‐micron particles were achieved through the topotactic reaction, which are optimal for homogeneous fluoride‐ion insertion.^[^ ^24^ ^]^ The EDX also yielded a Sn:Ti ratio of 2.08(1):1, which agrees with the reported formula of Sn_2_TiO_4_. HAXPES measurements corroborate the absence of Ba 3*d*, F 1*s*, and Cl 2*p* signals in the sample (Figure S5). Moreover, core‐level HAXPES suggests a formally divalent oxidation state for Sn; Sn 3*d*_3/2_ and 3*d*_5/2_ signals appear at binding energies of 494.4 and 485.9 eV, respectively, in close agreement with core‐level binding energies of SnO^[^ ^33^ ^]^ (Figure 1d).^[^ ^31^, ^34^ ^]^

Sn^2+^ expresses stereochemically active 5*s* ^2^ lone pairs of electrons whereas Sn^4+^ has a [Kr]4*d* ^10^5*s* ^0^ electron configuration. Therefore, energy‐variant (2 and 5 keV) valence band HAXPES measurements can spotlight stereochemical activity of electron lone pairs since the photoemission cross‐section of *s* and *p* orbitals decay with a shallower slope as a function of incident excitation energy,^[^ ^33^ ^]^ as compared to *d* and *f* orbitals.^[^ ^35^ ^]^ As such, higher incident energies bring into focus states with greater *s*‐orbital character. The observed differences in intensity of the energy‐variant valence band spectra, plotted in Figure S6, arise because of the stereochemical activity of the Sn^2+^ lone pairs.^[^ ^36^ ^]^ Broad features that become pronounced at 5 keV excitation deep in the valence band in the range of 12 to 9 eV correspond to Sn 5*s*─O 2*p* bonding states, whereas the second pronounced feature in the range of 7 to 5 eV corresponds to antibonding lone‐pair Sn 5*s*─O 2*p* states.^[^ ^33^ ^]^ The energy positioning of the antibonding states reflects the Sn‐5*p*‐mediated stabilization of filled anti‐bonding Sn 5*s*─O 2*p* states, which is characteristic of the stereochemically active Sn centers in oxides.^[^ ^37^ ^]^ The strong stereochemical expression of the lone pairs as visualized in Figure 1c is a result of the small Δ*E*_s–p_ separation on the order of 1.2–1.8 eV. Analogous to the distorted litharge structure adopted by SnO, a 1D tunnel is stabilized as a result of lone‐pair repulsions along the *c*‐axis of Sn_2_TiO_4_.^[^ ^35^ ^]^

Interestingly, core‐level Ti 2*p*_3/2_ and 2*p*_1/2_ HAXPES spectra of Sn_2_TiO_4_, provided in Figure 1e, lie at a lower binding energy as compared to that of TiO_2_, which indicates that the Ti atoms in Sn_2_TiO_4_ are slightly reduced and are not entirely tetravalent.^[^ ^32^ ^]^ The residual electron density is likely a result of the delocalization of electron density from stereochemically active lone pairs on Sn at the valence band edge. The nominally reduced Ti centers are crucial to enable charge compensation for topochemical insertion of fluoride ions within Sn_2_TiO_4_.

Sn_2_TiO_4_ was treated with XeF_2_ to probe the ability of the material to accommodate fluoride‐ions as per Equation (3) as a proxy for electrochemical fluoridation^[^ ^38^ ^]^:

(3) $${\mathrm{Sn}}_{2}{\mathrm{TiO}}_{4}\left(s\right)+x\;{\mathrm{XeF}}_{2}\left({\mathrm{CH}}_{3}\mathrm{CN}\right)\to {\mathrm{Sn}}_{2}{\mathrm{TiO}}_{4}F_{2x}\left(s\right)+x\;\mathrm{Xe}\;\left(g\right)$$

The extent of reversibility of the fluoridation was also investigated by heating the XeF_2_‐treated Sn_2_TiO_4_, denoted as Sn_2_TiO_4_F*_x_*, at 375 °C for 60 min under flowing Ar/H_2_ gas as per Equation (4).

(4) $$2{\mathrm{Sn}}_{2}{\mathrm{TiO}}_{4}F_{x}\left(s\right)+xH_{2}\left(g\right)\;\to \;2{\mathrm{Sn}}_{2}{\mathrm{TiO}}_{4}\;\left(s\right)+2x\mathrm{HF}\;\left(g\right)\;$$

The effect of each treatment on the average crystal structure was investigated using powder X‐ray diffraction. The stacked powder diffractograms, provided in Figure 1a, indicate that the Sn_2_TiO_4_ structure was retained post‐treatment. The absence of new reflections in the powder diffractograms also indicates that treatment with both XeF_2_ and Ar/H_2_ did not result in the formation of an interphase product.^[^ ^39^ ^]^ Moreover, Rietveld refinements reveal a reversible 0.0573(1)% unit cell contraction upon fluoride‐ion insertion (Figure 1a). The unit cell contraction is observed as a result of competing expansions (2.24(7)%) and contractions (4.00(1)%) of the [SnO_3_] and [TiO_6_] polyhedra, respectively. Rietveld refinement also suggested that the fluoride‐ions are located on Wyckoff 16*i* (dashed lines in Figure 1b) with a fractional occupancy of 0.124(2), yielding a refined composition of Sn_2_TiO_4_F_0.496(8)_, which corresponds to ca. 0.5 mol. fluoride‐ion insertion per formula unit of the insertion host. The refinement statistics and the refined crystal structure data of inserted and de‐inserted Sn_2_TiO_4_F*_x_* are provided in Tables S3 and S4, and Tables S5 and S6, respectively.

Bulk topochemical fluoride‐ion insertion in Sn_2_TiO_4_ has been evidenced using element‐specific techniques with varying penetration depths such as XANES, HAXPES, and EDX. F K‐edge XANES shows a clear F 1*s* peak (Figure S7a). In contrast to X‐ray photoelectron spectroscopy (XPS), which primarily probes surface electronic structure, the high excitation energies accessible in HAXPES enable interrogation of the bulk electronic structure due to the higher penetration depths (≈ 10–30 nm).^[^ ^40^ ^]^ As a result, the core‐level F 1*s* HAXPES feature, seen in Figure 1f, not only indicates that fluoridation was successful, but also confirms not just surface but bulk fluoride‐ion insertion. Furthermore, the absence of the F 1s feature after defluoridation confirms the complete removal of fluoride ions and demonstrates the complete reversibility of the fluoridation process. Probing even deeper into the bulk (1–2 µm), the EDX spectrum (Figure S7b) of Sn_2_TiO_4_F*_x_* shows the clear presence of F K‐edge features and yields an overall 2.15(1) Sn: 1 Ti: 0.64(7) F ratio generally consistent with the extent of fluoridation determined from X‐ray scattering. Moreover, EDX mapping (Figure S7c) shows that fluorine incorporation occurs homogeneously across the particles, consistent with bulk fluoride‐ion insertion inferred from HAXPES.

### Redox Processes at Ti Centers

Fluoride‐ion insertion in Sn_2_TiO_4_ can proceed through two possible mechanisms: i) the insertion of fluoride‐ions in vacant interstitial positions along tunnels within the host or ii) the substitution of lattice oxygen with fluorine. These two pathways can be distinguished as anion insertion would result in Ti oxidizing toward a formally tetravalent state, whereas substitution to produce [TiO_5_F] polyhedra would result in Ti reduction toward a formally trivalent state.

**Figure 2:**
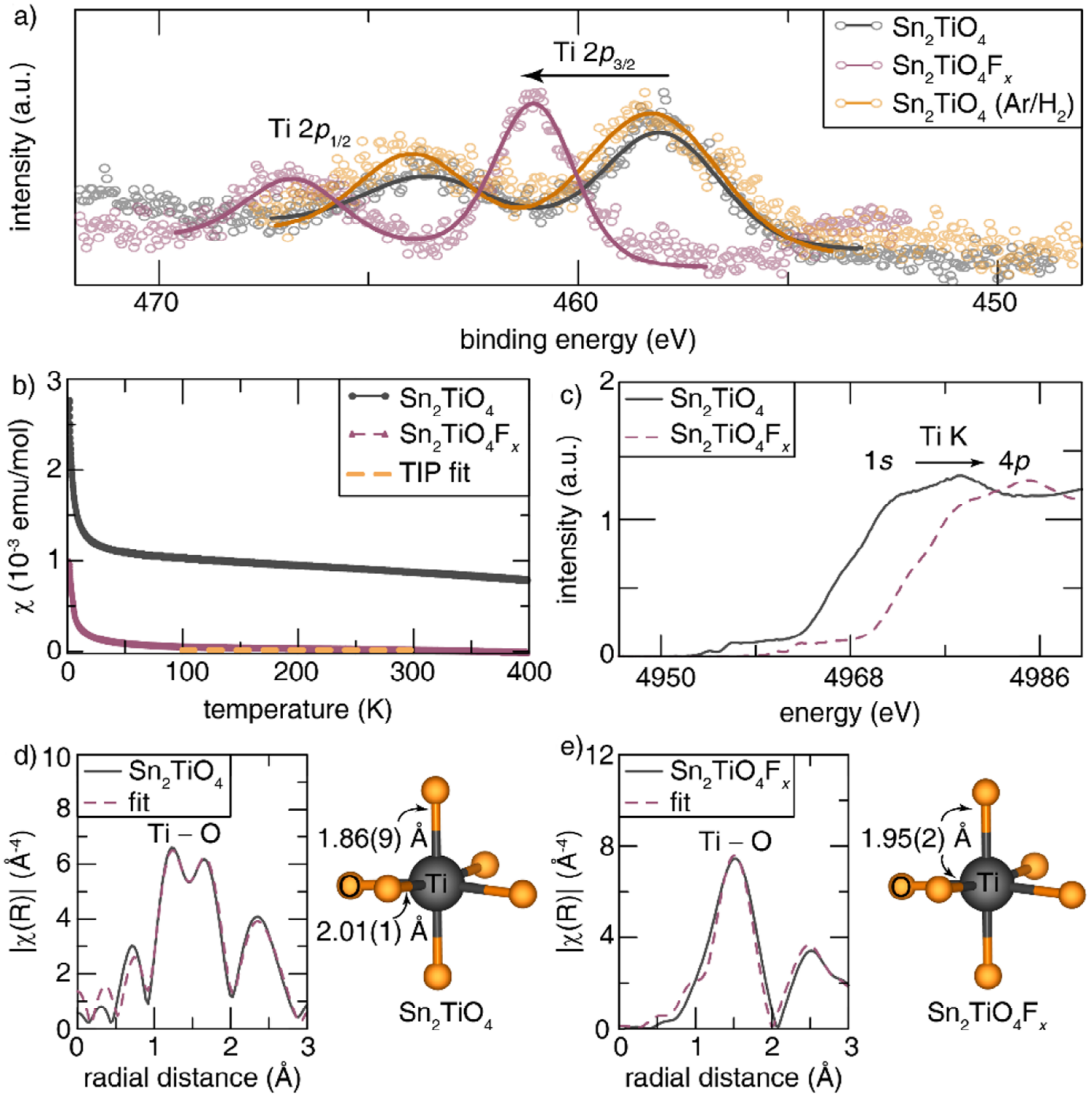
a) The insertion of fluoride‐ions is charge‐compensated through delocalized redox including the oxidation of Ti centers as evidenced by the Ti 2*p*_3/2_ and 2*p*_1/2_ peaks of Sn_2_TiO_4_F*_x_* (pink) shifting to higher binding energies relative to as‐prepared Sn_2_TiO_4_ (grey). The reversibility of fluoridation was also confirmed by the restoration of Ti 2*p*_3/2_ and 2*p*_1/2_ features for defluoridated Sn_2_TiO_4_ (orange) back to the same energies as measured for as‐prepared Sn_2_TiO_4_. The raw data is plotted as circles and the envelope of the fits are given as solid lines. b) Magnetic susceptibility^[^ ^45^ ^]^ measurements showing that Sn_2_TiO_4_ exhibits Curie Weiss‐like paramagnetic behavior, whereas Sn_2_TiO_4_F*_x_* exhibits essentially a temperature‐independent Pauli paramagnetism. The dotted orange line represents the fit for the temperature‐independent Pauli paramagnetic (TIP) background. c) The blue‐shift of the white‐line absorption in the Ti K‐edge XANES of Sn_2_TiO_4_F*_x_* (dashed, pink) relative to Sn_2_TiO_4_ (grey) can be attributed to Ti oxidation upon fluoridation. Fourier transforms of the EXAFS Ti K‐edge data (grey) and fit (dashed, pink) for d) Sn_2_TiO_4_ and e) Sn_2_TiO_4_F*_x_*. Ti─O bond lengths derived from the fits to the structural models are marked in the corresponding [TiO_6_] models where the Ti atom is grey and the O atoms are orange.

The oxidation state of Ti in Sn_2_TiO_4_, Sn_2_TiO_4_F*_x_*, and defluoridated Sn_2_TiO_4_ was first probed through core‐level Ti 2*p*_3/2_ and 2*p*_1/2_ HAXPES measurements. The Ti 2*p*_3/2_ and 2*p*_1/2_ core levels of Sn_2_TiO_4_ are shown as the grey trace in Figure 2a. Upon fluoridation, the Ti 2*p*_3/2_ and 2*p*_1/2_ peaks shift to higher binding energies relative to the pristine Sn_2_TiO_4_, suggesting that the Ti atoms have been oxidized.^[^ ^41^ ^]^ Defluoridation restores the Ti 2*p*_3/2_ and 2*p*_1/2_ peaks to that of pristine Sn_2_TiO_4_, indicating that Ti oxidation, and thus, the fluoridation of Sn_2_TiO_4_ is reversible.

Magnetic susceptibility measurements also corroborate the oxidation of the Ti centers to a tetravalent, 3*d* ^0^ state. Figure 2b shows field cooling temperature‐dependent magnetic susceptibility curves. Sn_2_TiO_4_ exhibits Curie−Weiss (CW)‐like paramagnetic behavior. The effective magnetic moment, obtained by performing a CW^[^ ^42^ ^]^ fit between 75 and 275 K, was determined to be 1.15 μ_B_ (Figure S8a). This value is less than the theoretical effective moment for Ti^3+^ in a d^1^ configuration (1.73 μB) but significantly larger than that of Ti^4+^ (0 μB). This indicates that the oxidation state in pristine Sn_2_TiO_4_ is between a formal tri‐ and tetra‐valent state, which enables oxidative anion insertion.^[^ ^43^ ^]^ Sn_2_TiO_4_F*_x_* exhibits a complex magnetic behavior characterized by diamagnetic features above 300 K and a dominant temperature‐independent Pauli paramagnetism (TIP) below this threshold with a χ_TIP_ value graphically estimated to be 5.56 × 10^−5^ emu·mol^−1^.^[^ ^44^ ^]^ Anion insertion results in a decrease in the magnetic susceptibility of Sn_2_TiO_4_F*_x_* relative to that of Sn_2_TiO_4_, as shown in Figure 2b.^[^ ^45^ ^]^ The upturn observed at low‐temperatures is attributed to the presence of impurity spins.^[^ ^44^ ^]^ The magnetization as a function of applied field was also measured at 2 K (Figure S8b). These data also reveal a decrease in the magnetization upon fluoridation, providing additional evidence for oxidation of Ti centers to a 3*d* ^0^ state.

The combination of the HAXPES and magnetism measurements suggests Ti oxidation, which would occur if fluoride‐ions were inserted within the Sn_2_TiO_4_ lattice. This was further supported by HAXPES, where the higher excitation energies allow the potential formation of oxygen vacancies within the bulk crystal structures to be probed. The O 1*s*, F 1*s*, and Sn 3*p*_3/2_ spectra of Sn_2_TiO_4_, Sn_2_TiO_4_F*_x_*, and defluoridated Sn_2_TiO_4_ spectra are provided in Figure S9. Each spectrum was normalized to the Sn 3*p*_3/2_ feature, allowing the intensities of the O 1*s* features of each sample to be compared. The O 1*s* intensity subtly increases for Sn_2_TiO_4_F*_x_* relative to Sn_2_TiO_4_, suggesting that oxygen vacancies were not generated, further supporting that fluoride‐ions were inserted into the Schafarzikite structure.

The Ti K‐edge XANES spectra of Sn_2_TiO_4_ and Sn_2_TiO_4_F*_x_* are plotted in Figure 2c. The spectra show a significant shift in the rising edge from 4962.87 (Sn_2_TiO_4_) to 4969.72 (Sn_2_TiO_4_F*_x_*) eV, suggestive of oxidative fluoride‐ion insertion. A subtle loss in intensity of the pre‐edge feature is also observed, suggesting a symmetrization of the [TiO_6_] octahedron (Figure S10).^[^ ^46^, ^47^ ^]^ Transforming the data to *R*‐space and fitting to models of local structure (Table S7) reveals significant differences between Sn_2_TiO_4_ (Figure 2d) and Sn_2_TiO_4_F*_x_* (Figure 2e). The *R*‐space EXAFS data of Sn_2_TiO_4_ has two prominent features located at a radial distance of ≈ 1.877 and 2.100 Å, corresponding to Ti─O_1_ and Ti─O_2_, respectively. These distinct correlations arise due to the Jahn‐Teller distorted sub‐tetravalent [TiO_6_] octahedra, which produce two distinct interactions of Ti with axial (O_1_) and equatorial (O_2_) oxygen atoms.^[^ ^48^ ^]^ Fitting the Ti─O coordination shells, shown in Figure 2d, yields Ti─O_1_ and Ti─O_2_ bond lengths of 1.893(4) and 2.031(2) Å, respectively. Comparing the *R*‐space Ti K‐edge EXAFS of Sn_2_TiO_4_ and Sn_2_TiO_4_F*_x_* reveals that the two distinct Ti─O correlations in Sn_2_TiO_4_ merge into a single coordination shell upon fluoridation. This suggests that the Jahn‐Teller distorted [TiO_6_] octahedra become symmetric upon fluoridation, which would occur when the electron from the *t*_2g_ orbital (considering a nominal 3*d* ^1^ electron configuration of Sn_2_TiO_4_) is lost upon Ti oxidation. This is consistent with a loss of residual electron density and oxidation of titanium centers as also attested to by the subtle loss in intensity of the pre‐edge feature upon fluoride‐ion insertion (Figure S10). This result is corroborated by fitting the first coordination shell of Sn_2_TiO_4_F*_x_*, which yielded a single Ti─O bond length of 1.952(5) Å (Figure 2e).

**Figure 3:**
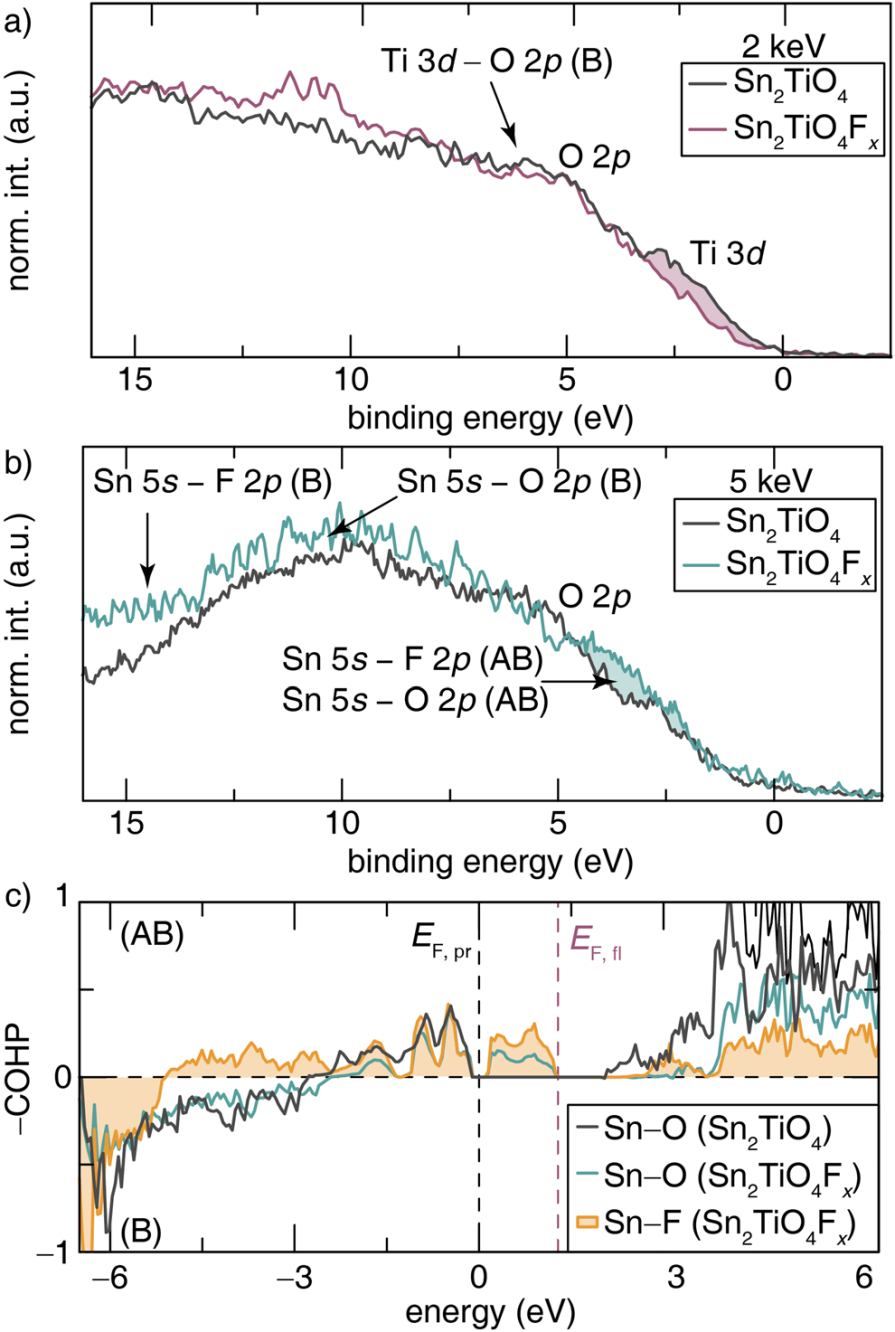
a) Valence band HAXPES spectra of Sn_2_TiO_4_ (grey) and Sn_2_TiO_4_F*_x_* (pink) acquired at 2 keV excitation energy. The differences in intensity at the valence band maximum shaded in pink are a result of Ti oxidation. b) Valence band spectra of Sn_2_TiO_4_ (grey) and Sn_2_TiO_4_F*_x_* (green) acquired at 5 keV excitation energy. The differences in intensity at the valence band maximum are shaded in green. c) Sn─O and Sn─F ─COHP analyses of Sn_2_TiO_4_ and Sn_2_TiO_4_F*_x_* reveal the creation of Sn─F and Sn─O antibonding states upon fluoride‐ion insertion. The dashed grey line indicated the Fermi level of Sn_2_TiO_4_ (*E*_F,pr_) and the dashed pink line (*E*_F,fl_) represents the Fermi level of Sn_2_TiO_4_F*_x_*. The Fermi level of Sn_2_TiO_4_F*_x_* was not set to 0 to preserve overlap of similar features between Sn_2_TiO_4_ and Sn_2_TiO_4_F*_x_*. (B) represents bonding interactions, whereas (AB) denotes anti‐bonding interactions.

The electronic structure of Sn_2_TiO_4_ and Sn_2_TiO_4_F*_x_* was first probed using energy‐variant valence band HAXPES. As noted above, the photoemission cross‐sections of the *s* and *p* orbitals decay slower at higher energies compared to *d* and *f* orbitals, which makes energy‐variant valence band HAXPES an excellent tool to spotlight the orbital contributions at the valence band maximum.^[^ ^33^, ^35^, ^49^ ^]^ As such, the valence band spectra collected at 2 keV can be used to understand the impact of fluoridation on Ti 3*d* states and its bonding interactions, whereas valence band spectra measured at 5 keV can be used to specifically spotlight Sn 5*s* ^2^ orbital contributions at the valence band maximum (Figure 3).^[^ ^36^ ^]^

The valence band spectra of Sn_2_TiO_4_ and Sn_2_TiO_4_F*_x_* acquired at an excitation energy of 2 keV are plotted in Figure 3a. The spectra were aligned to the O 2*p* feature present in both valence band spectra. The overlaid spectra delineate a notable decrease in intensity at the valence band edge upon fluoridation owing to a shift in the valence band of Sn_2_TiO_4_F*_x_* to higher binding energies. This can be attributed to the loss of states at the valence band maximum because of the oxidation of Ti. As a result, the calculated band offset between Sn_2_TiO_4_ and Sn_2_TiO_4_F*_x_* was determined to be 0.689 eV (Figure S11). The magnitude of the intensity decrease is consistent with oxidation from only partially reduced titanium centers and further corroborates the observed stoichiometry of ≈ 0.5 mols (Sn_2_TiO_4_F_0.496(8)_) of inserted fluoride ions per Sn_2_TiO_4_ formula unit.^[^ ^50^ ^]^

### The Formation of a Dative Bond Stabilizes the Inserted Fluoride‐ion

**Figure 4:**
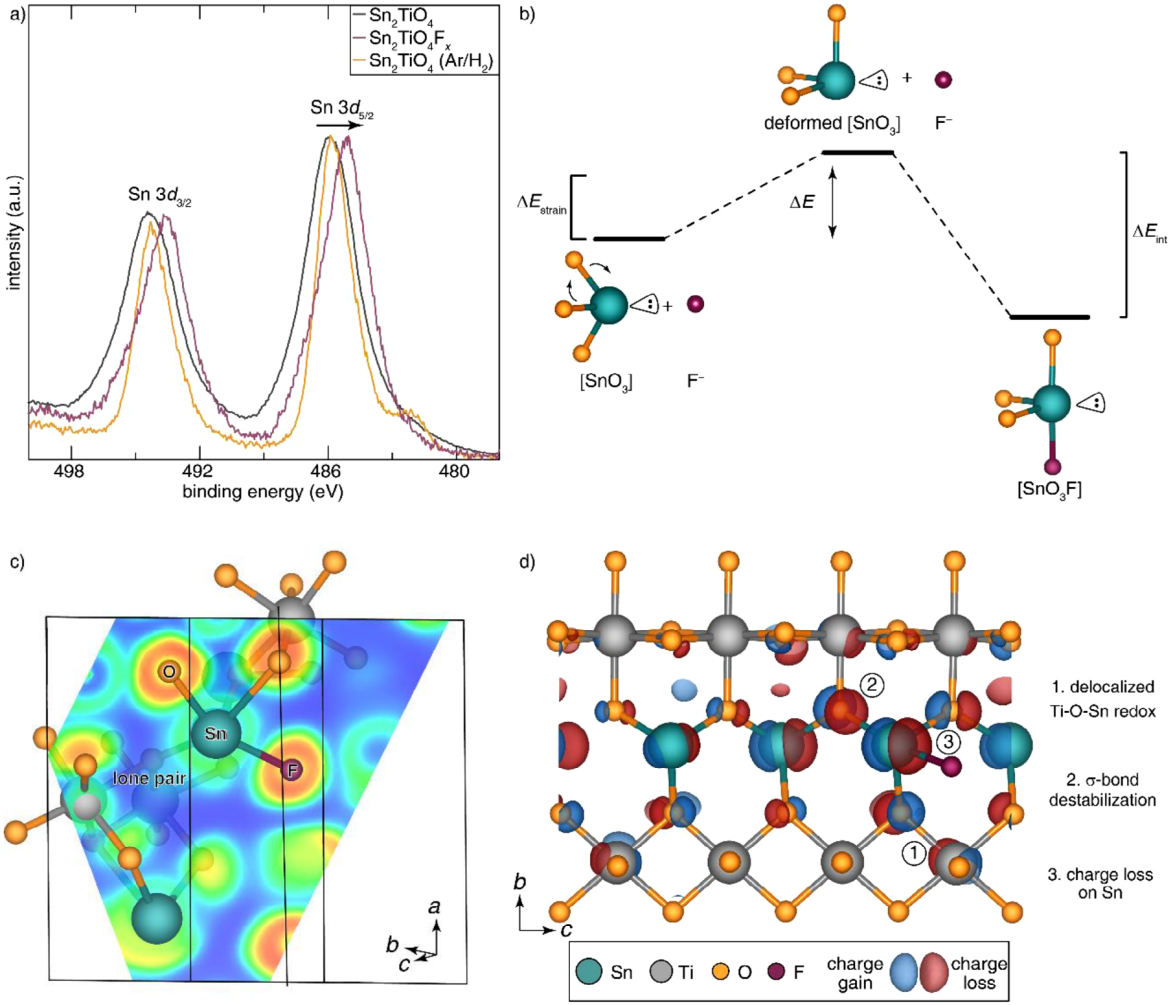
a) Core‐level Sn 3*d*_3/2_ and 3*d*_5/2_ HAXPES spectra of as‐prepared (grey), fluoridated (pink), and defluoridated (orange) Sn_2_TiO_4_ depict a subtle reduction in Sn upon fluoride‐ion insertion. b) A schematic depicting the mechanism of deformation and energetics involved with the formation of a dative Sn─F bond in Sn_2_TiO_4_. Adapted with permission from reference.^[^ ^54^ ^]^ c) The ELF of Sn_2_TiO_4_F*_x_* viewed along the (1.03135 1.33155 ‐1) plane to observe the Sn─F and Sn─O interactions in [SnO_3_F]. The ELF depicts covalent interactions between Sn and F. d) The charge difference density map of a 1 × 1 × 2 supercell Sn_2_TiO_4_F*_x_* with one fluoride ion inserted viewed along *a*.

Figure 4a shows that the Sn 3*d*_3/2_ and 3*d*_5/2_ core levels of Sn_2_TiO_4_F*_x_* lie at lower binding energies as compared to as‐prepared and defluoridated Sn_2_TiO_4_. This observed reduction of Sn is counterintuitive as fluoride‐ion insertion has been charge‐balanced through Ti oxidation. Therefore, the reduction of Sn is likely a result of the formation of a Sn─F bond between the Sn centers and fluoride‐ions situated within the 1D tunnels sketched in Figure 1b where the Sn centers gain electron density donated from the Lewis basic fluoride‐ion.

It is important to note that this Sn─F interaction bears the hallmarks of a dative bond, a closed‐shell interaction in which the fluoride acts as an electron donor toward the Lewis acidic Sn center.^[^ ^51^, ^52^ ^]^ Such a closed‐shell interaction involves the donation of the lone pair of electrons from the fluoride‐ion into the empty σ* orbital of Sn, while a coincident σ‐hole, situated along the vector (opposite) of a σ (Sn─O) bond, provides the necessary Coulombic stabilization to the newly formed bond.^[^ ^53^ ^]^ For this dative Sn─F bond to manifest, the local [SnO_3_] polyhedron of the Sn_2_TiO_4_ host would have to distort in order to minimize the inherent repulsion between the electron donating fluoride‐ion and the stereochemically active lone pairs of Sn^2+^. This deformation process, illustrated in Figure 4b, involves an inherent strain energy (∆*E*_strain_). The polarizability (lattice anharmonicity) of the stereochemically active lone pairs as the vertex of the pseudo‐tetrahedron yields the degree of structural flexibility necessary for the deformation that allows the rigid periodic crystal structure to remain intact. In the correct orientation, the [SnO_3_] pseudo‐tetrahedron contains a σ‐hole that allows the Sn to bond with the inserted fluoride‐ion directly opposite of the Sn─O σ‐bond, which manifests as an interaction energy (∆*E*_int_), which further represents the interplay between electrostatic forces (∆*V*_elstat_), orbital‐based interactions (∆*E*_oi_), and Pauli repulsions (∆*E*_Pauli_).^[^ ^54^ ^]^ It is important to note that this final optimized [SnO_3_F] polyhedron geometry is in line with the Galy‐Andersson model of a *sp* ^3^ *d* trigonal bipyramidal Sn.^[^ ^55^, ^56^ ^]^

We have further sought to understand the energetics and bonding interactions involved in the manifestation of the Sn─F dative bond through first‐principles DFT calculations. Geometry optimized structures are provided in Figure S12. The Sn_2_TiO_4_F*_x_* structure not only manifests a symmetrization of the [TiO_6_] octahedra, as observed from Ti K‐edge XANES (Figure S10) and EXAFS (Figure 2d,e), but also exhibits a 168° O─Sn─F bond vector, which is illustrative of the deformation of the [SnO_3_] pseudo‐tetrahedron (Figure 4b). A subtle, yet notable elongation of the Sn─O bond in the [SnO_3_F] polyhedron [Sn─O(1) = 2.212(9) Å] as compared to the [SnO_3_] pseudo‐tetrahedron [Sn─O(1) = 2.134(8) Å] in the starting compound is also observed. This elongated Sn─O(1) bond is coincident to the Sn─F [2.273(9) Å] vector, highlighting the Coulombic destabilization of the Sn─O interaction necessary for the formation of the Sn─F bond. It is important to note that these interactions, and particular, the nearly linear O─Sn─F interaction is somewhat reminiscent of a tetrel bond.^[^ ^57^ ^]^ However, tetrel interactions typically occur at distances significantly (typically > 0.5 Å) longer than the sum of the covalent radii of the two species.^[^ ^58^ ^]^ In contrast, the sum of the covalent radii of Sn and F is 1.96 Å, whereas the calculated Sn─F interaction is 2.273(9) Å, which is substantially shorter than a Sn─F tetrel bond (>2.46 Å). As such, the Sn─F interaction is best considered a dative bond. To the best of our knowledge, sigma‐hole interactions have not been considered thus far in deciphering the mechanistic origins of fluoride‐ion insertion in periodic solids, which represents a critical gap in the design of insertion hosts.

The combination of Sn─O bond destabilization and partial gain in electron density of Sn upon fluoride‐ion insertion implies a Lewis acid—base closed‐shell interaction. The magnitude of covalent (charge transfer) and electrostatic interactions occurring in this potential dative bond can be probed and visualized through ELF calculations and the construction of a charge density difference map (CDD), respectively. ELF provides a two‐or three‐dimensional, graphical representation of electron localization and ELF values (color coded and quantified where 0 = no localization, 0.5 = localization observed in a homogenous free electron gas, and 1 = perfect localization).^[^ ^59^, ^60^ ^]^ The high (>0.5) ELF value of the fluoride ion, low ELF value of Sn^2+^ (<0.5) and a small region with an approximate ELF value of 0 between the two atoms illustrates the electron delocalization from the fluoride‐ion, which directionally localizes around the Sn center. The large lobe of electron localization around the fluoride‐ion also suggests the donation of electrons to the Sn center to form the Sn─F bond (Figure 4c). In addition, the ELF highlights a covalent interaction between Sn and F atoms.^[^ ^61^ ^]^ This is in line with the majority of stannous fluorides, which also exhibit highly covalent Sn─F bonds.^[^ ^55^ ^]^ Furthermore, a CDD map along *a*, provided in Figure 4d, was constructed following Equation (5) where *ρ*Sn_2_TiO_4_F*_x_* (*r*), *ρ*Sn_2_TiO_4_(*r)*, and *ρ*F*_x_* (*r*) are the charge density maps of Sn_2_TiO_4_F*_x_*, Sn_2_TiO_4_, and a single fluoride‐ion, which was inserted into an empty 1 × 1 × 2 supercell of Sn_2_TiO_4_. The blue lobes represent gain in electron density, whereas the red lobes represent a loss in electron density.

(5) $$\Delta \rho \;\left(r\right)=\rho {\mathrm{Sn}}_{2}{\mathrm{TiO}}_{4}F_{x}\left(r\right)-\;\rho {\mathrm{Sn}}_{2}{\mathrm{TiO}}_{4}\left(r\right)-\;\rho F_{x}\left(r\right)$$

The CDD map features charge loss localized on the Ti atom adjacent to the inserted fluoride‐ion, highlighting the charge compensation mechanism. The second feature that can be observed in the CDD is the significant loss of charge around the O(1) atom that is coincident to the Sn─F dative bond, which is indeed larger in magnitude compared to the charge loss experienced by the remaining O atoms in the [SnO_3_F] polyhedron. This highlights the significant Sn─O σ‐bond destabilization that occurs to compensate for the formation of the dative bond. Further evidence for the dative bond can be observed as the prominent charge loss lobe on the Sn, which is a strong indicator of all of the electron density in the Sn─F bond being provided by the fluoride‐ion lone pairs. The CDD map, in this case, better captures the delocalized redox and dative bond formation as compared to formal Bader charge analyses.

Finally, a more detailed picture of the changes in the electronic structure upon dative bond formation can be obtained by measuring the valence band spectra of Sn_2_TiO_4_ before and after fluoride‐ion insertion at 5 keV and elucidating the specific orbital contributions through a corresponding crystal orbital Hamilton population (─COHP) analysis, which is a bond‐weighted density of states calculation to analyze interactions between pairs of atoms. The overlaid valence band spectra, shown in Figure 3b, depicts a subtle increase in states at the valence band maximum. This HAXPES feature is corroborated by the calculated ─COHP (Figure 3c) where the occupied electronic states seen at the top of the valence band are slightly higher for Sn_2_TiO_4_F*_x_* compared to the pristine Sn_2_TiO_4_ as a result of the weakening of the Sn─O bonds and the emergence of Sn─F bonds. The strength of hybridization is related again to Δ*E*_s—p_, which for Sn─F bonds will be greater than for Sn─O hybridization, but still significant, resulting in modified energy positioning of states at the valence band maximum.^[^ ^33^, ^49^ ^]^ COHP analyses further shows the emergence of new mid‐gap states at the top of the valence band, which derive from Sn 5*s* ─ F 2*p* antibonding interactions mediated by mixing with Sn 5*p* states as per the revised lone‐pair model.^[^ ^33^, ^49^ ^]^ The creation of Sn 5*s*,5*p* ─ F 2*p* anti‐bonding states is further indicative of the filling of the Sn σ* orbitals, as a result of formation of dative bonds.^[^ ^53^, ^54^ ^]^ Moreover, destabilization of the Sn─O(1) σ bond is further reflected in the appearance of new Sn─O antibonding midgap states. The filling of these Sn─O(1) antibonding states also has implications for O K‐edge XANES spectra (Figure S13). These spectra acquired for Sn_2_TiO_4_ exhibit two distinct pre‐edge features located at 529.40 and 532.09 eV. Upon fluoridation, only a single feature located at 530.74 eV is observed. The suppression of XANES features at low intensities is indicative of orbital filling as a result of Pauli blocking. Ultimately, the creation of new antibonding states at the valence band maximum with substantial Sn 5*s* character is what gives rise to the observed increased intensity of the Sn_2_TiO_4_F*_x_* valence band spectrum in Figure 3c. To further understand the differences in the Sn─O and Sn─F bonding interactions, the integrated COHP (iCOHP) values were calculated, which are a measure of the strength of covalency of a bond.^[^ ^62^ ^]^ The iCOHP of the Sn─O and Sn─F bonds were calculated to be −2.681 and −1.637, respectively. As a more negative iCOHP value indicates a stronger covalent bond, it can be understood that the Sn─O bonds are more covalent in nature compared to the Sn─F bonds.^[^ ^63^ ^]^ Notably, the overall weaker Sn─F bond as compared to Sn─O interactions is crucial for facilitating reversible fluoride‐ion diffusion through the tunnels of Sn_2_TiO_4_. As such, the combination of the formation of the Sn─F dative bond and the oxidation of Ti represent the thermodynamic driving force for fluoride‐ion insertion in Sn_2_TiO_4_, whereas the destabilizing repulsion from stereochemcially active lone pairs weakens lattice—ion interactions and enables bulk diffusion and reversibility.

### The Interplay Between Stereochemically Active Lone Pairs, Dative Bonds, and Delocalized Redox

The migration of ions through periodic solids involves collective processes that involve a local distortion of the host lattice and the interactions between the mobile ions and the host framework. cAIMD and first‐principles methods can struggle to capture the full complexity of ion diffusion such as the dynamic evolution of electron correlation, delocalized redox across Ti─O─Sn, and the coupling of rotational and vibrational modes, especially at timescales feasible for AIMD simulations. Despite these limitations, cAIMD still offers insights into fluoride‐ion migration and the energetics of diffusion barriers within Sn_2_TiO_4_. Ion diffusivity was calculated as per Equation (6) where *l* is the length of the jump, *v*_0_ is the jumping frequency, *n* is the dimensionality of the material, *E_a_* is the calculated diffusion energy barrier, *T* is the absolute temperature, and *D* is the diffusion coefficient.^[^ ^64^ ^]^

(6) $$D=\frac{l^{2}v_{0}}{2n}\;e^{-\frac{E_{a}}{\mathrm{kT}}}$$

Several possible diffusion pathways were identified and two were considered to be unique.^[^ ^65^ ^]^ A schematic of the lowest energy pathway, illustrated in Figure S14a, depicts fluoride‐ions moving as a screw (a translation and rotation) along the *c* direction. A calculated migration barrier of 530 meV and diffusivity of 3.93 × 10^−13^ cm^2^s^−1^ at 298 K (assuming a *v*_0_ of 10^13^ s^−1^) is modeled for the defect‐free periodic lattice (Figure S14b) but is likely lower in practice as a result of defects and non‐local concerted hopping mechanisms (such as occupancy conserved hopping)^[^ ^66^, ^67^ ^]^ not considered here (Video S1). The theoretical diffusion barrier obtained from the cAIMD simulations (performed for 1.5 fs) may be due to large extent of structural reorganization around Sn centers as the fluoride‐ion diffuses throughout the lattice. While there is inherent repulsion of the Sn 5*s* ^2^ and F stereochemically active lone pairs, the polarizability of the Sn 5*s* ^2^ electrons allows the Sn local coordination environment to evolve and locally distort in order to accommodate the fluoride‐ion.

## Conclusion

In summary, Sn_2_TiO_4_ with stereochemically active Sn 5*s* ^2^ electrons defining a 1D tunnel, prepared using a topotactic Sn exchange reaction, undergoes reversible fluoride‐ion (de)insertion up to a fluoride‐ion insertion stoichiometry of Sn_2_TiO_4_F_0.496(8)_. The results of the X‐ray spectroscopy, magnetic susceptibility measurements, and first‐principles calculations provide important insights into design principles for fluoride‐ion insertion electrodes. Taken in tandem, fluoride‐ion insertion and diffusion is mediated by the interplay between formation of Sn─F dative interactions and redox processes delocalized across Ti─O─Sn hybrid states counterbalanced by repulsions between fluoride‐ions and polarized Sn 5*s* ^2^ stereochemically active lone pairs. The coupled ion—electron transfer and transport processes are mediated along large 1D tunnels that themselves result from stereochemical expression and repulsions between Sn 5*s* ^2^ electron lone pairs. To the best of our knowledge, the full range of anion—lattice interactions available for fluoride‐ions in periodic solids from tetrel interactions to covalent dative bonds, which are significantly more complex as compared to alkali‐ and alkaline‐earth ions, have not thus far been considered. Modulating such interactions will be a critical design rule in the development of new fluoride‐ion electrodes, which must be counterbalanced with stereochemical expression of 4/5/6*s* ^2^ lone pairs to provide weak interactions that allow for facile fluoride‐ion migration between interstitial sites. In turn, the thermodynamic driving force can be tuned through modulation of the redox processes to access higher redox potentials and multi‐electron redox. Ensuring more facile ion diffusion will require site‐selective modification to weaken dative interactions, modulate stereochemical activity, and expand bottlenecks to cation diffusion. Specific insertion patterns within individual particles can be probed through single‐particle mapping of fluoride‐ion insertion using X‐ray ptychography and tomography.

## Conflict of Interests

The authors declare no conflict of interest.

## Supporting information



Supporting Information

Video S1

## Data Availability

The data that support the findings of this study are available from the corresponding author upon reasonable request.

## References

[1] M. S. Whittingham , Science 1976, 192, 1126–1127. 17748676 10.1126/science.192.4244.1126

[2] A. Sood , A. D. Poletayev , D. A. Cogswell , P. M. Csernica , J. T. Mefford , D. Fraggedakis , M. F. Toney , A. M. Lindenberg , M. Z. Bazant , W. C. Chueh , Nat. Rev. Mater. 2021, 6, 847–867.

[3] J. Kim , H. Kim , K. Kang , Adv. Energy Mater. 2018, 8, 1702646.

[4] F. Wu , G. Yushin , Energy Environ. Sci. 2017, 10, 435–459.

[5] Y. Li , Y. Lu , P. Adelhelm , M.‐M. Titirici , Y.‐S. Hu , Chem. Soc. Rev. 2019, 48, 4655–4687. 31294739 10.1039/c9cs00162j

[6] Y. Preger , L. Torres‐Castro , J McDowall , *U.S. DOE Energy Storage Handbook*, Department of Energy 2013, pp. 1–17.

[7] M. A. Nowroozi , S. Ivlev , J. Rohrer , O. Clemens , J. Mater. Chem. A 2018, 6, 4658–4669.

[8] S. T. Hartman , R. Mishra , J. Mater. Chem. A 2020, 8, 24469–24476.

[9] M. A. Nowroozi , I. Mohammad , P. Molaiyan , K. Wissel , A. R. Munnangi , O. Clemens , J. Mater. Chem. A 2021, 9, 5980–6012.

[10] W. Zaheer , J. L. Andrews , A. Parija , F. P. Hyler , C. Jaye , C. Weiland , Y.‐S. Yu , D. A. Shapiro , D. A. Fischer , J. Guo , J. M. Velázquez , S. Banerjee , ACS Energy Lett. 2020, 5, 2520–2526.

[11] J. L. Andrews , E. T. McClure , K. K. Jew , M. B. Preefer , A. Irshad , M. J. Lertola , D. D. Robertson , C. Z. Salamat , M. J. Brady , L. F. J. Piper , S. H. Tolbert , J. Nelson Weker , B. F. Chmelka , B. S. Dunn , S. R. Narayan , W. C. West , B. C. Melot , ACS Energy Lett. 2022, 7, 2340–2348.

[12] P. Gao , M. A. Reddy , X. Mu , T. Diemant , L. Zhang , Z. Zhao‐Karger , V. S. K. Chakravadhanula , O. Clemens , R. J. Behm , M. Fichtner , Angew. Chem. Int. Ed. 2016, 55, 4285–4290. 10.1002/anie.201509564 26924132

[13] X. Zhao , Z. Zhao‐Karger , D. Wang , M. Fichtner , Angew. Chem. Int. Ed. 2013, 52, 13621–13624. 10.1002/anie.201307314 24346944

[14] X. Zhao , S. Ren , M. Bruns , M. Fichtner , J. Power Sources 2014, 245, 706–711.

[15] M. Anji Reddy , M. Fichtner , J. Mater. Chem. 2011, 21, 17059.

[16] A. W. Xiao , G. Galatolo , M. Pasta , Joule 2021, 5, 2823–2844.

[17] D. T. Thieu , M. H. Fawey , H. Bhatia , T. Diemant , V. S. K. Chakravadhanula , R. J. Behm , C. Kübel , M. Fichtner , Adv. Funct. Mater. 2017, 27, 1701051.

[18] D. Zhang , K. Yamamoto , A. Ochi , Y. Wang , T. Yoshinari , K. Nakanishi , H. Nakano , H. Miki , S. Nakanishi , H. Iba , T. Uchiyama , T. Watanabe , K. Amezawa , Y. Uchimoto , J. Mater. Chem. A 2021, 9, 406–412.

[19] D. Zhang , K. Yamamoto , Z. Cao , Y. Wang , Z. Zhong , H. Kiuchi , T. Watanabe , T. Matsunaga , K. Nakanishi , H. Miki , H. Iba , Y. Harada , K. Amezawa , K. Maeda , H. Kageyama , Y. Uchimoto , J. Am. Chem. Soc. 2025, 147, 5649–5657. 39804710 10.1021/jacs.4c12391

[20] N. H. Bashian , M. Zuba , A. Irshad , S. M. Becwar , J. Vinckeviciute , W. Rahim , K. J. Griffith , E. T. McClure , J. K. Papp , B. D. McCloskey , D. O. Scanlon , B. F. Chmelka , A. Van der Ven , S. R. Narayan , L. F. J. Piper , B. C. Melot , Chem. Mater. 2021, 33, 5757–5768.

[21] D. A. Santos , S. Rezaei , D. Zhang , Y. Luo , B. Lin , A. R. Balakrishna , B.‐X. Xu , S. Banerjee , Chem. Sci. 2023, 14, 458–484. 36741524 10.1039/d2sc04157j PMC9848157

[22] B. P. de Laune , G. J. Rees , M. J. Whitaker , H.‐Y. Hah , C. E. Johnson , J. A. Johnson , D. E. Brown , M. G. Tucker , T. C. Hansen , F. J. Berry , J. V. Hanna , C. Greaves , Inorg. Chem. 2017, 56, 594–607. 27977159 10.1021/acs.inorgchem.6b02466

[23] B. P. de Laune , G. J. Rees , J. F. Marco , H.‐Y. Hah , C. E. Johnson , J. A. Johnson , F. J. Berry , J. V. Hanna , C. Greaves , Inorg. Chem. 2017, 56, 10078–10089. 28776991 10.1021/acs.inorgchem.7b01613

[24] W. Zaheer , G. Agbeworvi , S. Perez‐Beltran , J. L. Andrews , Y. Aierken , C. Weiland , C. Jaye , Y.‐S. Yu , D. A. Shapiro , S. C. Fakra , D. A. Fischer , J. Guo , D. Prendergast , S. Banerjee , Cell Rep. Phys. Sci. 2021, 2, 100592.

[25] H. Giefers , F. Porsch , G. Wortmann , Solid State Ionics 2005, 176, 199–207.

[26] S. Ohara , H. Takizawa , Y. Hayashi , Chem. Lett. 2010, 39, 364–365. (accessed 9/10/2024).

[27] J. Boltersdorf , I. Sullivan , T. L. Shelton , Z. Wu , M. Gray , B. Zoellner , F. E. Osterloh , P. A. Maggard , Chem. Mater. 2016, 28, 8876–8889.

[28] S. O'Donnell , A. Hamilton , P. A. Maggard , J. Electrochem. Soc. 2019, 166, H3084–H3090.

[29] E. A. Gabilondo , R. J. Newell , R. Broughton , A. Koldemir , R. Pöttgen , J. L. Jones , P. A. Maggard , Angew. Chem. Int. Ed. 2024, 63, e202312130. 10.1002/anie.202312130 37699142

[30] S. O'Donnell , A. Smith , A. Carbone , P. A. Maggard , Inorg. Chem. 2022, 61, 4062–4070. 35192323 10.1021/acs.inorgchem.1c03846

[31] M. A. Stranick , A. Moskwa , Surf. Sci. Spectra 1993, 2, 45–49 (accessed 9/10/2024).

[32] C. Rath , P. Mohanty , A. C. Pandey , N. C. Mishra , J. Phys. D: Appl. Phys. 2009, 42, 205101.

[33] S. A. Razek , M. R. Popeil , L. Wangoh , J. Rana , N. Suwandaratne , J. L. Andrews , D. F. Watson , S. Banerjee , L. F. J. Piper , Electronic Structure 2020, 2, 023001.

[34] W. Xia , H. Wang , X. Zeng , J. Han , J. Zhu , M. Zhou , S. Wu , CrystEngComm 2014, 16, 6841–6847.

[35] A. Walsh , D. J. Payne , R. G. Egdell , G. W. Watson , Chem. Soc. Rev. 2011, 40, 4455. 21666920 10.1039/c1cs15098g

[36] L. A. H. Jones , W. M. Linhart , N. Fleck , J. E. N. Swallow , P. A. E. Murgatroyd , H. Shiel , T. J. Featherstone , M. J. Smiles , P. K. Thakur , T.‐L. Lee , L. J. Hardwick , J. Alaria , F. Jäckel , R. Kudrawiec , L. A. Burton , A. Walsh , J. M. Skelton , T. D. Veal , V. R. Dhanak , Phys. Rev. Mater. 2020, 4, 074602.

[37] A. Walsh , G. W. Watson , J. Phys. Chem. B 2005, 109, 18868–18875. 16853428 10.1021/jp051822r

[38] C. K. Blakely , S. R. Bruno , S. K. Kraemer , A. M. Abakumov , V. V. Poltavets , J. Solid State Chem. 2020, 289, 121490.

[39] A. R. Giem , J. R. Ayala , J. Cheng , C. Weiland , C. Jaye , D. A. Fischer , S. Banerjee , Chem. Commun. 2024, 60, 14589–14592. 10.1039/d4cc04331f 39569674

[40] S. Siol , J. Mann , J. Newman , T. Miyayama , K. Watanabe , P. Schmutz , C. Cancellieri , L. P. H. Jeurgens , Surf. Interface Anal. 2020, 52, 802–810.

[41] U. Diebold , T. E. Madey , Surf. Sci. Spectra 1996, 4, 227–231 (accessed 9/10/2024).

[42] P. Weiss , J. Phys. Theor. Appl. 1907, 6, 661–690.

[43] C. H. Perry , D. L. Kinser , L. K. Wilson , J. G. Vaughn , J. Appl. Phys. 1979, 50, 1601–1603 (accessed 3/25/2025).

[44] J.‐G. Cheng , T. Ishii , H. Kojitani , K. Matsubayashi , A. Matsuo , X. Li , Y. Shirako , J.‐S. Zhou , J. B. Goodenough , C. Q. Jin , M. Akaogi , Y. Uwatoko , Phys. Rev. B 2013, 88, 205114.

[45] J. H. Van Vleck , in The Theory of Electric and Magnetic Susceptibilities, Oxford University Press, Oxford 1932.

[46] J. Li , Y. Li , P. K. Routh , E. Makagon , I. Lubomirsky , A. I. Frenkel , J. Synchrotron Radiat. 2021, 28, 1511–1517. 34475298 10.1107/S1600577521007025

[47] Medium: X. F. Farges , G. E. Brown , J. J. Rehr , Geochim. Cosmochim. Acta 1996, 60, 3023–3038.

[48] A. Demourgues , L. Gautier , A. V. Chadwick , C. Delmas , Nucl. Instrum. Methods Phys. Res. B 1997, 133, 39–44.

[49] J. V. Handy , W. Zaheer , A. R. M. Rothfuss , C. R. McGranahan , G. Agbeworvi , J. L. Andrews , K. E. García‐Pedraza , J. D. Ponis , J. R. Ayala , Y. Ding , D. F. Watson , S. Banerjee , Chem. Mater. 2022, 34, 1439–1458.

[50] R. Dronskowski , P. E. Bloechl , J. Phys. Chem. 1993, 97, 8617–8624.

[51] A. Nandi , S. Kozuch , Chem. ‐ Eur. J. 2020, 26, 759–772. 31536146 10.1002/chem.201903736

[52] O. Hassel , C. Rømming , Q. Rev. Chem. Soc. 1962, 16, 1–18.

[53] L. T. Maltz , F. P. Gabbaï , Inorg. Chem. 2023, 62, 13566–13572. 37551938 10.1021/acs.inorgchem.3c01987 PMC10862541

[54] B. L. Murphy , F. P. Gabbaï , J. Am. Chem. Soc. 2023, 145, 19458–19477. 37647531 10.1021/jacs.3c06991 PMC10863067

[55] G. Dénès , M. C. Madamba , H. Merazig , A. Muntasar , Z. Zhu , AIP Conf. Proc. 2016, 1781, 020006, 10.1063/1.4966002 (accessed: March 2025).

[56] J. Galy , G. Meunier , S. Andersson , A. Åström , J. Solid State Chem. 1975, 13, 142–159.

[57] W.‐C. Liu , F. P. Gabbaï , Science 2024, 385, 1184–1188. 39265017 10.1126/science.adp7465

[58] A. Bauzá , S. K. Seth , A. Frontera , Coord. Chem. Rev. 2019, 384, 107–125.

[59] A. Savin , R. Nesper , S. Wengert , T. F. Fässler , Angew. Chem. Int. Ed. Eng. 1997, 36, 1808–1832.

[60] P. Fuentealba , E. Chamorro , J. C. Santos , in Theoretical and Computational Chemistry, Vol. 19 (Ed: A. Toro‐Labbé ), Elsevier, Amsterdam 2007, pp. 57–85.

[61] A. C. Legon , Phys. Chem. Chem. Phys. 2017, 19, 14884–14896. 28561824 10.1039/c7cp02518a

[62] M. Khazaei , J. Wang , M. Estili , A. Ranjbar , S. Suehara , M. Arai , K. Esfarjani , S. Yunoki , Nanoscale 2019, 11, 11305–11314. 31165851 10.1039/c9nr01267b

[63] L. Guo , G. Tang , J. Hong , Chin. Phys. Lett. 2019, 36, 056201.

[64] M. Okubo , Y. Tanaka , H. Zhou , T. Kudo , I. Honma , J. Phys. Chem. B 2009, 113, 2840–2847. 19708215 10.1021/jp8099576

[65] A. Liivat , J. O. Thomas , Solid State Ionics 2011, 192, 58–64.

[66] Y. He , E. Scivally , A. Shaji , B. Ouyang , Y. Zeng , Adv. Energy Mater. 2025, 15, 2403877.

[67] J. Cheng , M. Udayakantha , S. Perez‐Beltran , L. Carrillo , W. Zaheer , L. Zuin , S. Banerjee , CrystEngComm 2024, 26, 5165–5176.

