# Supplementary material for: Interplay Between Stereochemically Active Lone Pair Repulsions, Sigma Hole Interactions, and Delocalized Redox Processes in Topochemical Fluoride‐Ion Insertion
Source: Angew Chem Int Ed Engl. 2025 Jun 23;64(33):e202507650. doi: 10.1002/anie.202507650 (PMC12338408; doi:10.1002/anie.202507650)
Supplement: Supplementary file 1 — Supporting Information [file ANIE-64-e202507650-s002.docx]

**Interplay between Stereochemically Active Lone Pair Repulsions, Sigma Hole Interactions, and Delocalized Redox Processes in Topochemical Fluoride-Ion Insertion**

**Supplementary Information**

Anindya Pakhira^†,1^, Shruti Hariyani^†,1^, George Agbeworvi^1^, Jaime R. Ayala^1^, Conan Weiland^2^, Cherno Jaye^2^, Daniel A. Fischer^2^, Lu Ma^3^, and Sarbajit Banerjee*^,1,4,5^

^1^Department of Chemistry, Texas A&M University, College Station, TX, 77843, USA

^2^Material Measurement Laboratory, National Institute of Standards and Technology, Gaithersburg, MD 20899, USA

^3^National Synchrotron Light Source II, Brookhaven National Laboratory, Upton, NY, 11973, USA

^4^Laboratory for Inorganic Chemistry, Department of Chemistry and Applied Biosciences, ETH Zurich, Vladimir-Prelog-Weg 2, CH-8093 Zürich, Switzerland
^5^Laboratory for Battery Science, PSI Center for Energy and Environmental Sciences, Paul Scherrer Institute, Forschungsstrasse 111, CH-5232 Villigen PSI, Switzerland

E-mail: [sbanerje@ethz.ch](mailto:sbanerje@ethz.ch)

*corresponding author; ^†^these authors contributed equally^†^

**Methodology**

Synthesis and Topochemical Fluoride-Ion Insertion: Sn_2_TiO_4_ was obtained through a topotactic ion-exchange reaction with Ba_2_TiO_4_. The precursors BaCO_3_ (Strem Chemicals, 99.9%) and TiO_2_ (Strem Chemicals, 99+%) were combined in a stoichiometric ratio, hand ground with an agate mortar and pestle, and further mixed for 20 min using a high-energy ball mill (SPEX SamplePrep 8000D mill using ½ˈˈ×1ˈˈ polystyrene vials and yttria-stabilized zirconia 3.0 mm diameter balls purchased from Advanced Materials). The mixture was then transferred into an alumina crucible (MSE PRO, 50×40×30 mm), which was placed in a programmable KSL-1200X muffle furnace and heated to 900°C for 2 h with a heating and cooling rate of 7°C/min. The reaction was quenched and the resulting powder was re-ground, pressed into a 6 mm pellet and heated twice, first at 900°C for 1 h, and then again at 1100°C for 4 h, using a heating and cooling rate of 7.3°C/min^1^.

The obtained Ba_2_TiO_4_ (**Figure S1**) showed excellent agreement with the published crystal structure (ICSD #2625) and was subsequently mixed with a SnClF peritectic mixture, which was prepared by mixing SnCl_2_ (Sigma Aldrich, 99%) and SnF_2_ (Sigma Aldrich, 99%) with Ba_2_TiO_4_ in a 1.1:1.1:1 ratio for 20 min in an Ar-filled glovebox maintained at O_2_ and H_2_O levels of <0.1 ppm and <0.1 ppm, respectively. The mixture was then transferred into a fused-silica tube, which was then evacuated and flame sealed. The tubes were heated to 400°C in a programmable box furnace at a rate of $\approx$13°C/min for 24 h and then allowed to radiatively cool to room temperature. The final product was ground and then washed and centrifuged with deionized water (prepared using a GenPure UV/UF xCAD plus) to remove the Ba-containing side products. The product was then allowed to dry overnight under ambient conditions.

The following preparatory methods did not yield phase-pure Sn_2_TiO_4_. A direct solid-state synthesis route was explored using stoichiometric quantities of TiO_2_ (Strem Chemicals, 99+%) and SnO (Sigma-Aldrich, 99%) in a 1:2 molar ratio. The precursors were thoroughly hand ground in an agate mortar and pestle for 15 minutes to ensure homogeneity. The resulting powder mixture was transferred into an alumina crucible and heated to 600°C (Thermo Scientific Lindberg Blue M tube furnace) for 24 h under flowing Ar. Phase analysis of the reaction products using X-ray diffraction analysis (Figure S1) revealed the formation of SnO_2_ and β-Sn_0.875_, indicating disproportionation of the SnO precursor, along with the presence of unreacted anatase TiO_2_. To promote enhanced reactivity, the synthesis was repeated under identical conditions but at an elevated temperature of 800°C. However, the same disproportionation products (SnO_2_ and β-Sn_0.875_) were obtained accompanied by the presence of rutile TiO_2_.

Upon obtaining Sn_2_TiO_4_, we turned to fluoride-ion insertion reagents, which enable evaluation of putative fluoride-ion insertions hosts without need for identification of a compatible electrolyte and the construction of full cells. This process is analogous to the use of alkyllithium compounds, such as *n*-BuLi, by Whittingham, which accelerated the discovery and development of Li-ion battery cathode materials.^2^ Fluoridation of Sn_2_TiO_4_ was performed by stirring 0.244 g Sn_2_TiO_4_ with a 3-fold molar excess (0.355 g) of XeF_2_ (Thermo Fisher, 99.5%) in 30 mL of dry acetonitrile (Thermo Fisher, 99.9%) at 23°C in a 100 mL round bottom flask.^3, 4^

XeF_2_ was chosen as our fluoridation reagent because of the entropic driving force towards based on the release of Xe gas, which further ameliorates the formation of solid by-products. XeF_2_ has also been successful in the fluoridation of FeSb_2_O_4,_ Bi_2_PdO_4_, and Bi_1.6_Pb_0.4_PtO_4_.^4, 5^ Moreover, these reactions can be conducted at ambient temperature and pressures, which represents a challenge for other fluoridating agents such as AgF, AgF_2_, and poly(vinyldene fluoride). Poltavets et al. have used XeF_2_ to prepare SrCoO_2.5_F_0.5_ as well as to synthesize two new oxyfluorides, La_4_Ni_3_O_8_F_1_ and La_4_Ni_3_O_8_F_2_ based on F-ion insertion into brownmillerite-related parent structures.^6^ In a typical reaction, the dispersion was stirred under flowing Ar under Schlenk conditions for 24 h at ambient temperatures. The reaction was then quenched, the excess acetonitrile decanted, and the obtained product was washed and centrifuged three times with 50 mL acetonitrile.^3, 4^ The powder was subsequently dried and stored under Ar.

De-fluoridation was performed by heating Sn_2_TiO_4_F*_x_* at 375°C (Thermo Scientific Lindberg Blue M tube furnace) for 1 h under flowing 95% Ar/ 5%H_2_  (v/v) gas.^3, 4^ Warning! This reaction generates HF gas and should therefore be properly vented in a fumehood.

**Materials Characterization:** Powder X-ray diffraction data were collected in Bragg-Brentano geometry using a Bruker AXS D8 ADVANCE ECO A25 diffractometer and Cu Kα radiation (λ = 1.5418 Å; 40 kV voltage, 25 mA current). All Rietveld refinements were performed using the Fullprof suite software^7^. The Vesta III software suite was utilized to create all the crystal structure representations shown in this article.^8^

Scanning electron microscopy (SEM) and energy dispersive X-ray (EDX) microscopy was conducted using a JEOL JSM-7500 field-emission instrument, which comprises an ultrahigh-resolution field emission scanning electron microscope equipped with a high brightness conical field-emission gun, a low-aberration conical objective lens, and an Oxford EDS spectrometer. The accelerating voltage was fixed at 20 KeV. Polycrystalline powders of the pristine, fluoridated, and defluoridated Sn_2_TiO_4_ were affixed to the surface of conductive carbon tape prior to imaging. Each sample was sputter coated using a 208HRD High Resolution Sputter Coater (Ted Pella) equipped with a Rotary-Planetary-Tilting RPT Stage and MTM-20 Thickness Controller. The target is a Pt/Pd target and the thickness was set to 5 nm.

Hard X-ray photoelectron spectroscopy (HAXPES) measurements were performed at the National Institute of Standards and Technology beamline SST-2 of National Synchrotron Light Source II at Brookhaven National Lab at excitation energies of 2 keV and 5 keV. All of the data were collected with a pass energy of 500 eV and a step size of 0.05 eV with the analyzer axis oriented parallel with the photoelectron polarization vector. ^9^. A double crystal monochromator allowed for photon energy selection; Si (111) crystals were used for 2 keV photon energy and Si (220) crystals were used for 5 keV. The beam energy was calibrated to the Fermi level of a gold foil prior to measurement. The collected HAXPES spectra were energy calibrated to carbon at 284.8 eV to ensure accurate binding energies for the collected core levels and valence bands.

Magnetic measurements of Sn_2_TiO_4_ and Sn_2_TiO_4_F*_x_* were conducted using a Quantum Design magnetic property measurement system using the Quantum Design superconducting quantum interference device (SQUID) magnetometer option. Both zero-field cooled (ZFC) and field-cooled (FC) measurements were performed from 2—400 K with an applied field up to 0.1 T. Field-dependent magnetization measurements were performed at 2K and above room temperature under an applied magnetic field ranging from –7 to +7 T.

The local structure environments of pristine and fluoridated Sn_2_TiO_4_ were probed by measuring the Ti K-edge using extended X-ray absorption fine structure spectroscopy (EXAFS)^10^ at the National Synchrotron Light Source II of Brookhaven National Laboratory Quick X-ray Absorption and Scattering beamline 7-BM. The samples were affixed to poly(4,4'-oxydiphenylene-pyromellitimide) tape (Kapton) and loaded into the sample holder. 25 scans of each edge were measured and subsequently averaged to improve the signal-to-noise ratio. Experiments were performed in ambient conditions. Prior to measurement, the beam was calibrated to metal foils. Both fluorescence and transmittance signals were collected using a Canberra PIPS detector and Pilatus3 S 900K (5-31 keV), respectively. Athena from the IFEFFIT package was used for further data sanitization. The data were aligned to a Ti foil and the data in the k range 3 – 12.5 Å^-1^ was Fourier transformed to obtain R-space data, which was used for shell fitting using the Artemis program from the IFEFFIT software package. The photoelectron mean free path, scattering amplitudes, and phase functions were calculated using the FEFF6 program. The initial model used for the EXAFS fitting was obtained from the Rietveld refinements.

XANES measurements were collected in partial electron yield (PEY) mode at beamline SST-1 of the National Synchrotron Light Source II at Brookhaven National Laboratory operated by the National Institute of Standards and Technology. A detector entrance grid bias of −300 V was used to reduce the low-energy electrons and improve the surface sensitivity. Charging was minimized by utilizing a charge compensation gun. The O K-edge was measured using a resolution of 0.2 eV. The PEY signals were normalized to the incident beam intensity using the photocurrent from a freshly evaporated gold mesh and the obtained spectra were energy calibrated to the O K-edge of a standard TiO_2_ sample.

**Computational simulations**: Total energy electronic structure calculations were performed within the framework of density functional theory (DFT) as implemented in the Vienna *ab initio* simulation package, using periodic boundary conditions to represent the solid. The initial model optimizations were performed using a projected augmented wave (PAW)^11^ methodology with a plane-wave basis set expansion of up to 520 eV^12^ and a generalized gradient approximation using the Perdew-Burke-Ernzerhof functional (GGA-PBE)^13^ to account for the electronic exchange and correlation to model electron-ion interactions for all the calculations^14^. The presence of partially occupied fluorine sites observed from Rietveld refinements (Sn_2_TiO_4_F_0.496(8)_) necessitated the creation of 1×1×2 supercells where every permutation of fluorine ordering was modeled and optimized. The final supercells corresponded to Sn_2_TiO_4_F_0.5_. The lowest energy configuration was employed for all further calculations. A Γ-point centered reciprocal grid of 6×6×4 points^15^ was used for relaxation of the supercell structures. All structural optimizations implemented an electronic convergence criterion set to 1E^-6^ eV and an ionic convergence criterion set to 1E-4 eV/Å**.** The density of states for both the host and the fluoride-ion-inserted structures were computed using the Heyd-Scuseria-Erzenhof^16^ screen hybrid exchange and correlation functional, HSE06. This functional incorporates a 75%:25% combination of PBE:Hartree-Fock, with a range separation of 0.2Å, which serves to address the considerable underestimation of the band gap when calculated using the PBE functional alone. The projected density of states (pDOS) was utilized to calculate the crystal orbital Hamilton population (COHP) using the local orbital suite toward electronic-structure reconstruction (LOBSTER) package^17^. The absolute charge spilling is lower than 1.81% for both the host and the fluoridated compound.

The energy diffusion barrier was obtained using constrained *ab initio* molecular dynamics simulations (cAIMD).^18^ In each simulation, a 1×1×3 supercell was modeled, and the lowest energy configuration was utilized. A plane-wave energy cutoff of 520 eV with a minimal Γ-centered 1×1×1 *k*-point mesh was adopted. The initial structure was fully relaxed at 0K and no framework melting was observed in all the simulations. The cAIMD method calculates the free energy change by performing a thermodynamic integration of the free-energy gradient while the system is being constrained onto the reaction path through the SHAKE algorithm^19^ in conjunction with the Blue Moon ensemble method for free energy calculations. The cAIMD simulations were performed by employing the Verlet algorithm which integrates Newton’s equations during the AIMD simulations performed with the NVT ensemble. The temperature was set to 298K using the Nosé-Hoover thermostat, which was selected to examine the probability of room temperature diffusion. The simulations were performed with a time step of 1.5 fs.

| **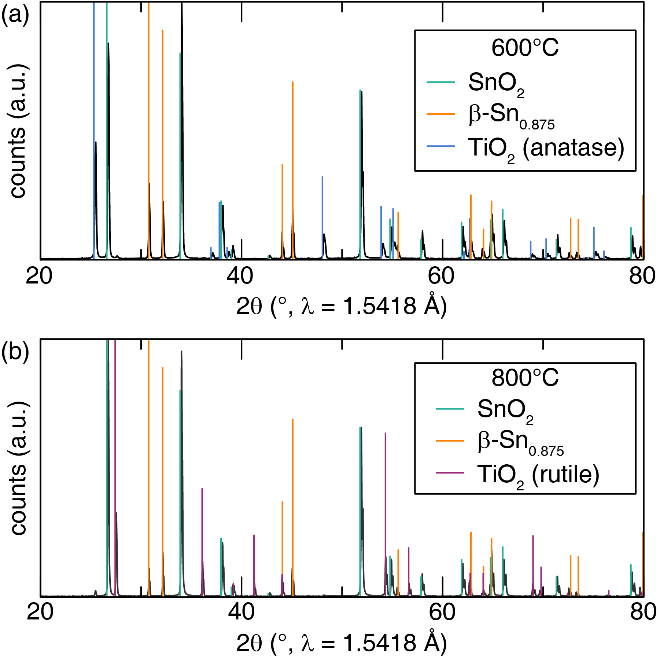** |
| --- |
| **Figure S1.** Results of direct solid-state reactions attempting to obtain Sn_2_TiO_4_ based on the reaction of SnO and TiO_2_ (anatase) in a 2:1 (mol/mol) ratio. (a) Sintering precursors at 600°C yields a mixture of anatase TiO_2_.and disproportion products of SnO_2_ and ϐ-Sn_0.875_. (b) Annealing the same precursor mixture at 800°C yielded SnO_2_, ϐ-Sn_0.875_, and rutile TiO_2_. |

| 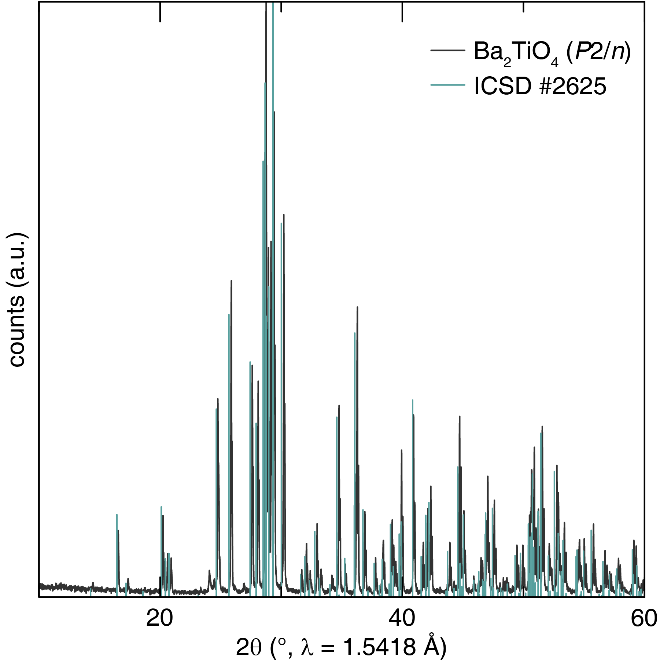 |
| --- |
| **Figure S2.** The powder X-ray diffractogram of Ba_2_TiO_4_ shows excellent agreement with the published crystal structure (ICSD #2625). |

| 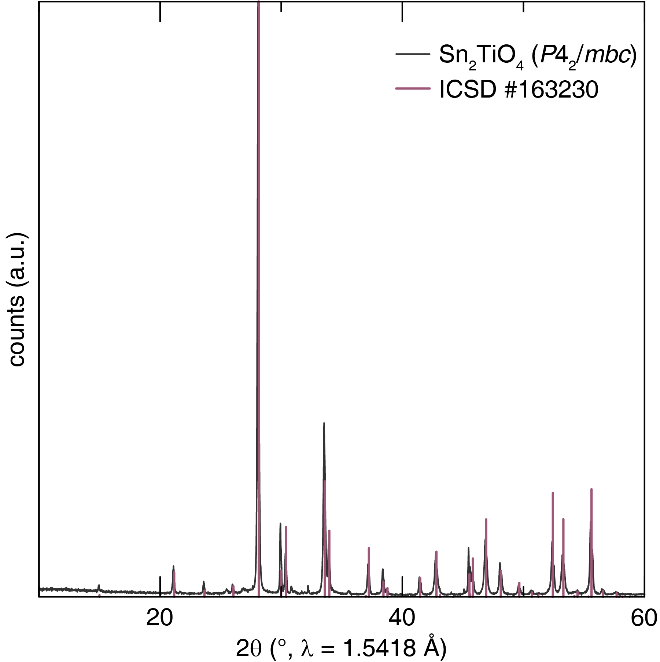 |
| --- |
| **Figure S3.** Powder X-ray diffractogram of Sn_2_TiO_4_ obtained from the topochemical treatment of Ba_2_TiO_4_ with the SnCl_2_ and SnF_2_ peritectic salt mixture indexed against ICSD #163230. |

| **Table S1.** Structure refinement parameters obtained though Rietveld refinements of as-prepared Sn_2_TiO_4_. | |
| --- | --- |
| Formula | Sn_2_TiO_4_ |
| Radiation type, λ (Å) | Cu Kα – radiation, 1.5418 |
| 2Θ range (deg) | 5—90 |
| Temperature (K) | 295 |
| Crystal system | Tetragonal |
| Space group, *Z* | *P*4_2_/*mbc*; 4 |
| Lattice parameters (Å) | *a* = *b* = 8.492(8) *c* = 5.923(7) |
| Volume (Å^3^) | 427.1316(0) |
| R_p_ (%) | 4.1 |
| R_wp_ (%) | 6.7 |
| R_exp_ (%) | 3.38 |
| χ^2^ | 3.97 |

| **Table S2.** Refined atom positions, thermal parameters, and occupancies as obtained from refinement of the powder XRD pattern for Sn_2_TiO_4_. | | | | | | |
| --- | --- | --- | --- | --- | --- | --- |
| atom | Wyck. pos. | *x* | *y* | *z* | *U*iso (Å^2^) | occ. |
| Sn1 | 8*h* | 0.1449(6) | 0.1643(6) | 0 | 0.033(1) | 1 |
| Ti1 | 4*d* | 0 | ½ | ¼ | 0.023(4) | 1 |
| O1 | 8*g* | 0.6761(0) | 0.1761(0) | ¼ | 0.027(3) | 1 |
| O2 | 8*h* | 0.0873(0) | 0.6035(0) | 0 | 0.010(2) | 1 |

| 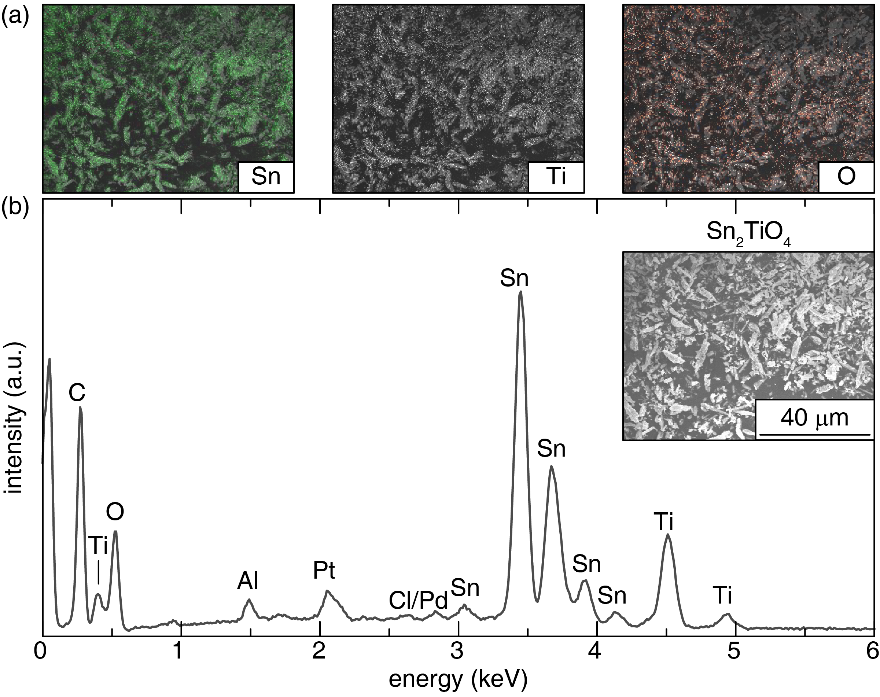 |
| --- |
| **Figure S4. (a)** EDX maps obtained for as-prepared Sn_2_TiO_4_. **(b)** The EDX spectrum of Sn_2_TiO_4_ shows no observable trace of F and Ba. The observed Al, C, and Pt/Pd signals are a consequence of the sample holder, carbon tape, and the sputtering process, respectively. An inset of the corresponding SEM image is also provided. |

| 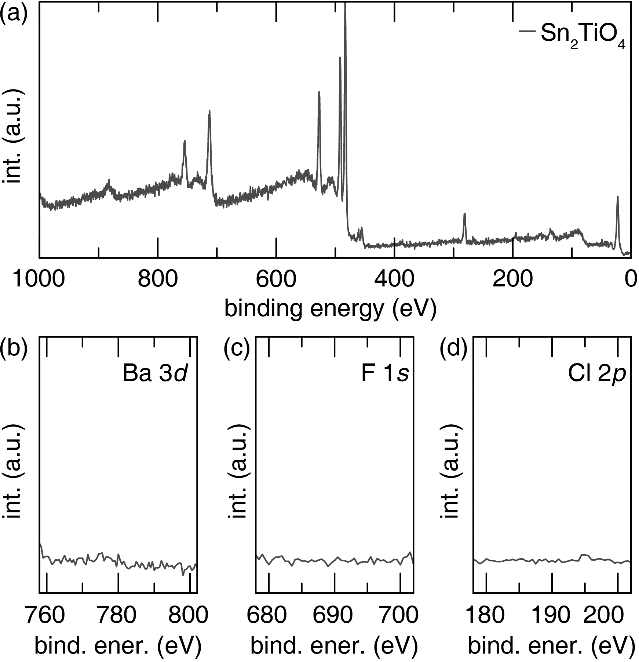 |
| --- |
| **Figure S5. (a)** The survey HAXPES scan of pristine Sn_2_TiO_4_. The sample shows the successful removal of BaCl_2_ and BaF_2_ with no discernible signal within limits of detection from **(b)** Ba 3*d*, **(c)** F 1*s*, and **(d)** Cl 2*p*. |

| 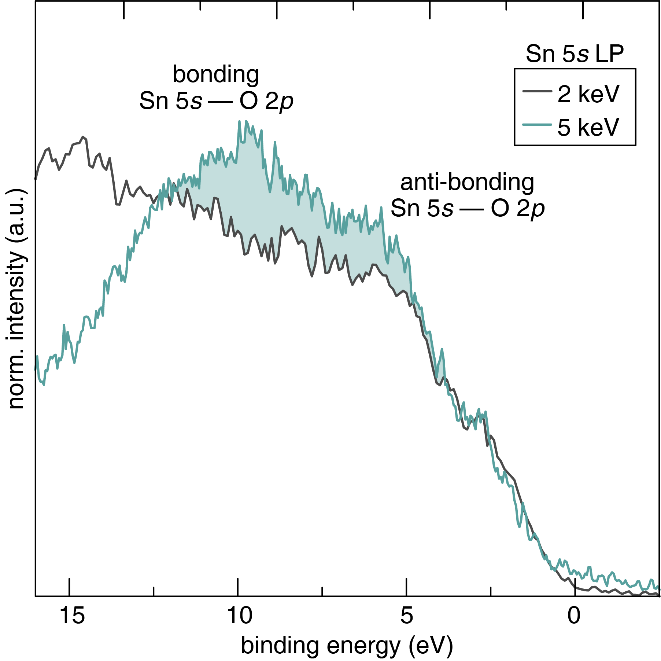 |
| --- |
| **Figure S6.** Valence band HAXPES spectra of Sn_2_TiO_4_ acquired at 2 keV (grey) and 5 keV (green) excitation. The intensity differences are shaded green for clarity. The observed increase in intensity is derived from the larger photoemission cross-sections of the 5*s* orbitals at higher kinetic energies. The broad pronounced features deep in the valence band in the range of -12 to -9 eV correspond to Sn 5*s—*O 2*p* bonding states, whereas the second pronounced feature in the range of -7 to -5 eV corresponds to the antibonding lone-pair Sn 5*s*—O 2*p* state. |

| **Table S3.** Structure refinement parameters obtained though Rietveld refinements of Sn_2_TiO_4_F*_x_*. | |
| --- | --- |
| Formula | Sn_2_TiO_4_F_0.0496(8)_ |
| Radiation type, λ (Å) | Cu Kα – radiation, 1.5418 |
| 2Θ range (deg) | 5—90 |
| Temperature (K) | 295 |
| Crystal system | Tetragonal |
| Space group, *Z* | *P*4_2_/*mbc*; 4 |
| Lattice parameters (Å) | *a* = *b* = 8.491(1) *c* = 5.921(8) |
| Volume (Å^3^) | 426.8868(1) |
| R_p_ | 7.5 |
| R_wp_ | 13.1 |
| R_exp_ | 5.01 |
| χ^2^ | 6.9 |

| **Table S4.** Refined atom positions, thermal parameters, and occupancies of Sn_2_TiO_4_F*_x_*. | | | | | | |
| --- | --- | --- | --- | --- | --- | --- |
| atom | Wyck. pos. | *x* | *y* | *z* | *U*iso (Å^2^) | occ. |
| Sn1 | 8*h* | 0.1462(0) | 0.1619(0) | 0 | 0.034(1) | 1 |
| Ti1 | 4*d* | 0 | ½ | ¼ | 0.026(2) | 1 |
| O1 | 8*g* | 0.6860(0) | 0.1861(0) | ¼ | 0.025(1) | 1 |
| O2 | 8*h* | 0.0720(0) | 0.5900(0) | 0 | 0.012(3) | 1 |
| F1 | 16*i* | 0.5030(0) | 0.6021(0) | 0.2341(0) | 0.043(2) | 0.124(2) |

| **Table S5.** Structure refinement parameters obtained though Rietveld refinements of defluoridated Sn_2_TiO_4_ (Ar/H_2_). | |
| --- | --- |
| formula | Sn_2_TiO_4_ |
| Radiation type, λ (Å) | Cu Kα – radiation, 1.5418 |
| 2Θ range (deg) | 5—90 |
| Temperature (K) | 295 |
| Crystal system | Tetragonal |
| Space group, *Z* | *P*4_2_/*mbc*; 4 |
| Lattice parameters (Å) | *a* = *b* = 8.492(3) *c* = 5.924(1) |
| Volume (Å^3^) | 427.2037(1) |
| R_p_ | 5.0 |
| R_wp_ | 10.9 |
| R_exp_ | 4.71 |
| χ^2^ | 5.35 |

| **Table S6.** Refined atom positions, thermal parameters, and occupancies of defluoridated Sn_2_TiO_4_ (Ar/H_2_). | | | | | | |
| --- | --- | --- | --- | --- | --- | --- |
| atom | Wyck. pos. | *x* | *y* | *z* | *U*iso (Å^2^) | occ. |
| Sn1 | 8*h* | 0.1440(0) | 0.1637(0) | 0 | 0.027(1) | 1 |
| Ti1 | 4*d* | 0 | ½ | ¼ | 0.025(2) | 1 |
| O1 | 8*g* | 0.6787(0) | 0.1787(0) | ¼ | 0.021(1) | 1 |
| O2 | 8*h* | 0.0813(0) | 0.6052(0) | 0 | 0.015(2) | 1 |

| *_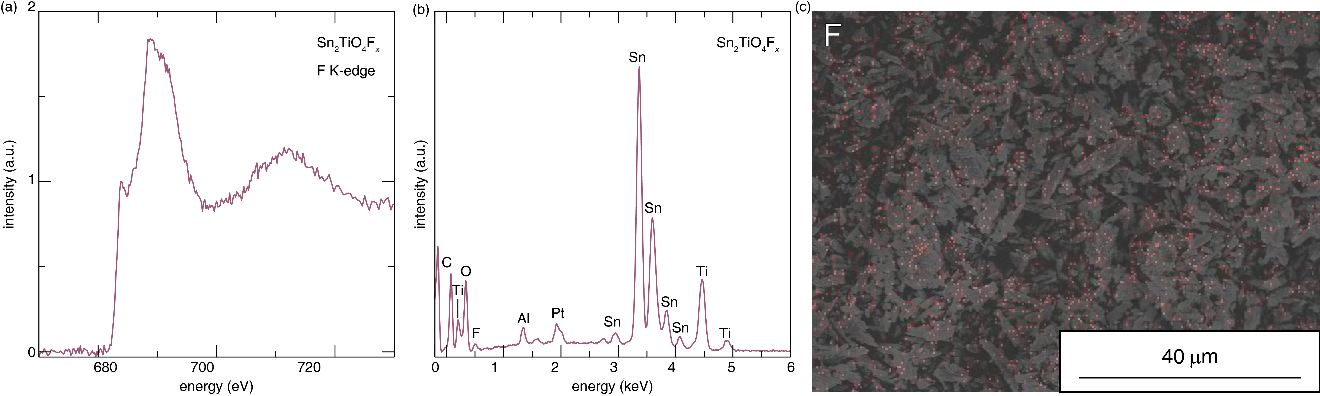_* |
| --- |
| **Figure S7. (a)** F K-edge XANES and **(b)** EDX spectrum and **(c)** F map of Sn_2_TiO_4_F*_x_* indicate the homogeneous insertion of fluoride-ions within Sn_2_TiO_4_ particles.   \| 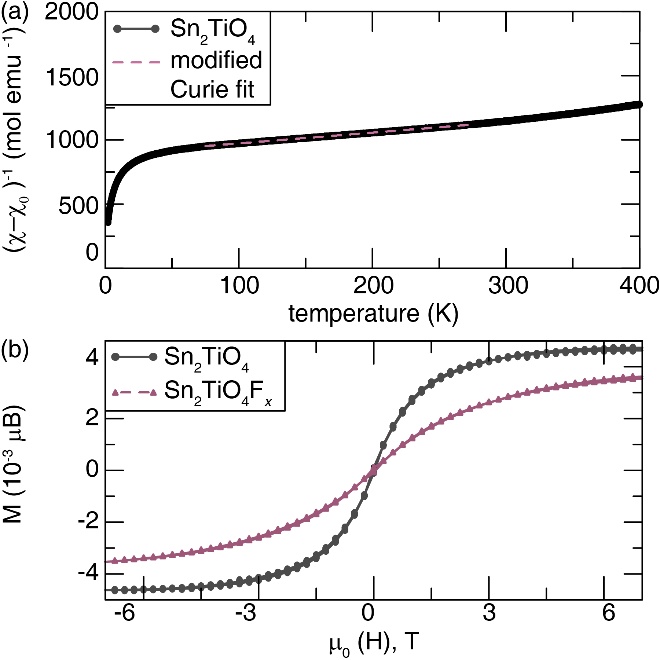 \| \| --- \| \| **Figure S8. (a)** Inverse magnetic susceptibility data of Sn_2_TiO_4_ (grey) from 2 – 400 K and the modified Curie-Weiss fit (dashed pink). **(b)** Magnetization versus magnetic field plotted for Sn_2_TiO_4_ (pink) and Sn_2_TiO_4_F*_x_* (grey) at 2K. \| |

| 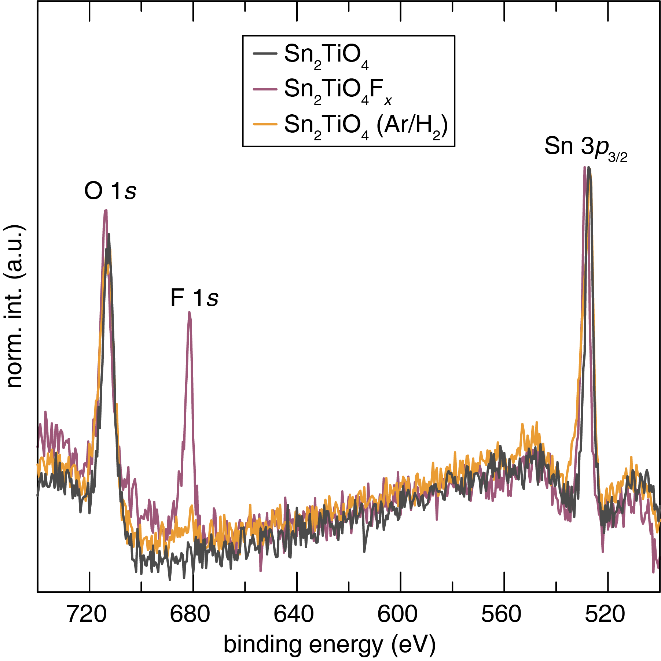 |
| --- |
| **Figure S9.** HAXPES O 1*s*, F 1*s*, and Sn 3*p*_3/2_ spectra for Sn_2_TiO_4_ (grey), Sn_2_TiO_4_F*_x_* (pink), and defluoridated Sn_2_TiO_4_ (orange). The data were normalized to the Sn 3*p*_3/2_ feature. The O 1*s* feature of each of the samples are comparable. A subtle increase in the O 1*s* intensity of Sn_2_TiO_4_F*_x_* is observed, which suggests that F-ion incorporation into the Sn_2_TiO_4_ lattice does not occur via fluorine substituting for lattice oxygen. |

| 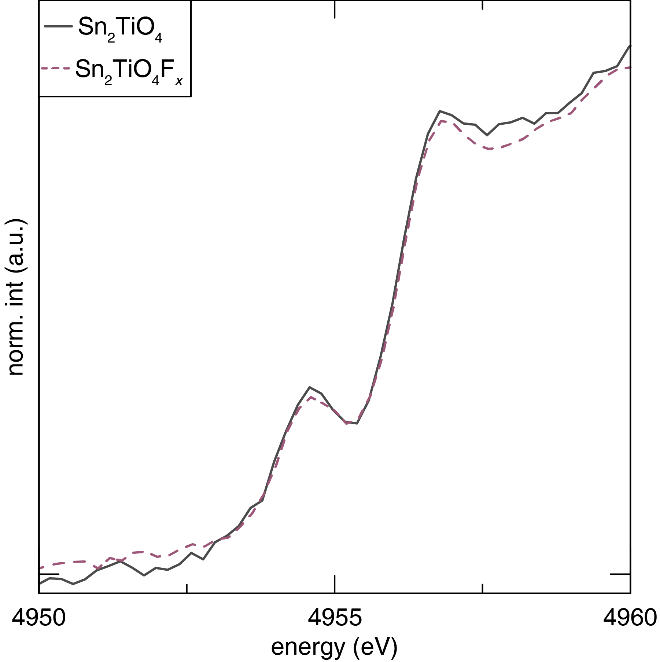 |
| --- |
| **Figure S10.** Magnified view of the pre-edge region of Ti K-edge XANES spectra of Sn_2_TiO_4_ (grey) and Sn_2_TiO_4_F*_x_* (dashed pink) depicts a subtle decrease in intensity, which suggests that the [TiO_6_] octahedra become more symmetric upon fluoride-ion insertion. |

| **Table S7.** Results of fitting EXAFS data to structural models of [TiO_6_] octahedra for as-prepared and fluoridated Sn_2_TiO_4_. | | | | | | |
| --- | --- | --- | --- | --- | --- | --- |
|  | Path | R (Å) | σ^2^ (Å^2^) | $S_{0}^{2}$ | ∆E_0_ (eV) | R-factor |
| Sn_2_TiO_4_ | Ti-O_axial_ | 1.886(4) | 0.0005(3) | 0.946 | -26.711 | 1.5% |
|  | Ti-O_equatorial_ | 2.022(6) | 0.0042(6) | 0.946 | -26.711 | 1.5% |
| Sn_2_TiO_4_F*_x_* | Ti-O_axial_ | 1.977(2) | 0.0051(2) | 0.850 | -9.600 | 1.4% |
|  | Ti-O_equatorial_ | 1.977(2) | 0.0051(2) | 0.850 | -9.600 | 1.4% |

| 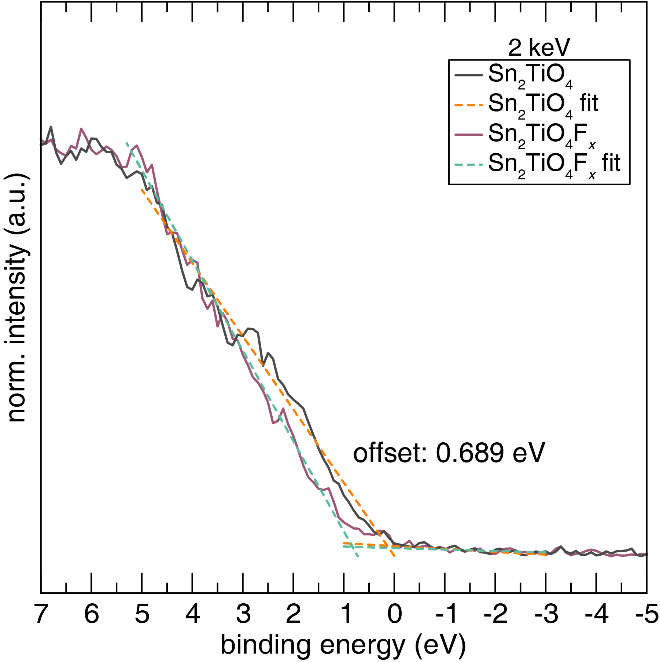 |
| --- |
| **Figure S11.** Fitting the valence band maxima of Sn_2_TiO_4_ (grey) and Sn_2_TiO_4_F*_x_* (pink) yields a band offset value of 0.689 eV. |

| 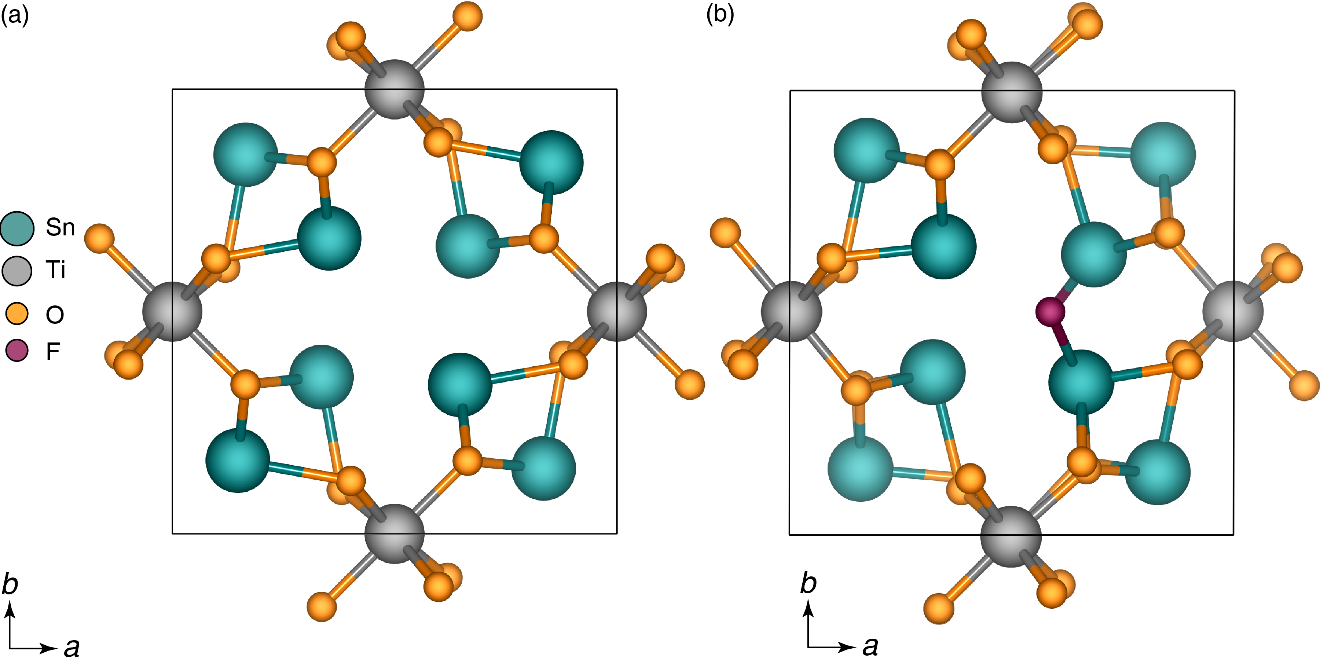 |
| --- |
| **Figure S12.** DFT-optimized crystal structures of **(a)** Sn_2_TiO_4_ and **(b)** Sn_2_TiO_4_F*_x_*. |

| 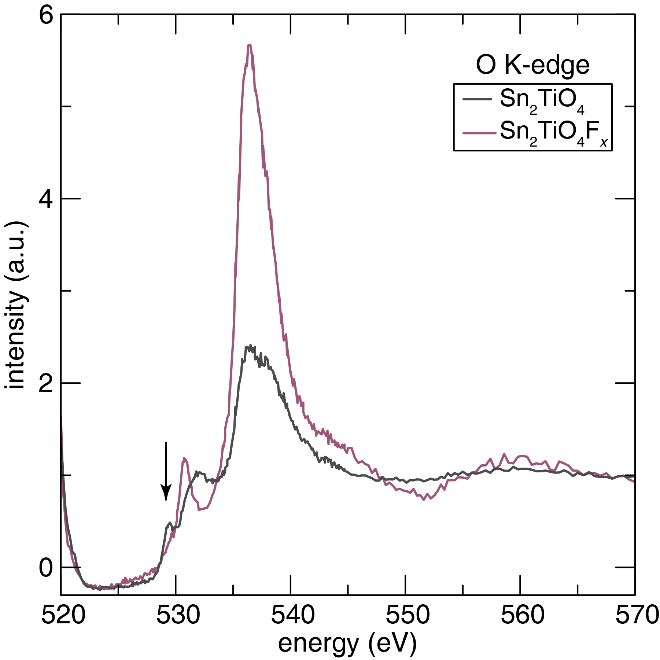 |
| --- |
| **Figure S13.** The O K-edge XANES spectrum of Sn_2_TIO_4_ (grey) shows two distinct pre-edge features, whereas the O K-edge XANES of Sn_2_TiO_4_F*_x_* (pink) shows only one pre-edge feature. This provides further indication that fluoridation fills an O 2*p*-centered antibonding states. |

| 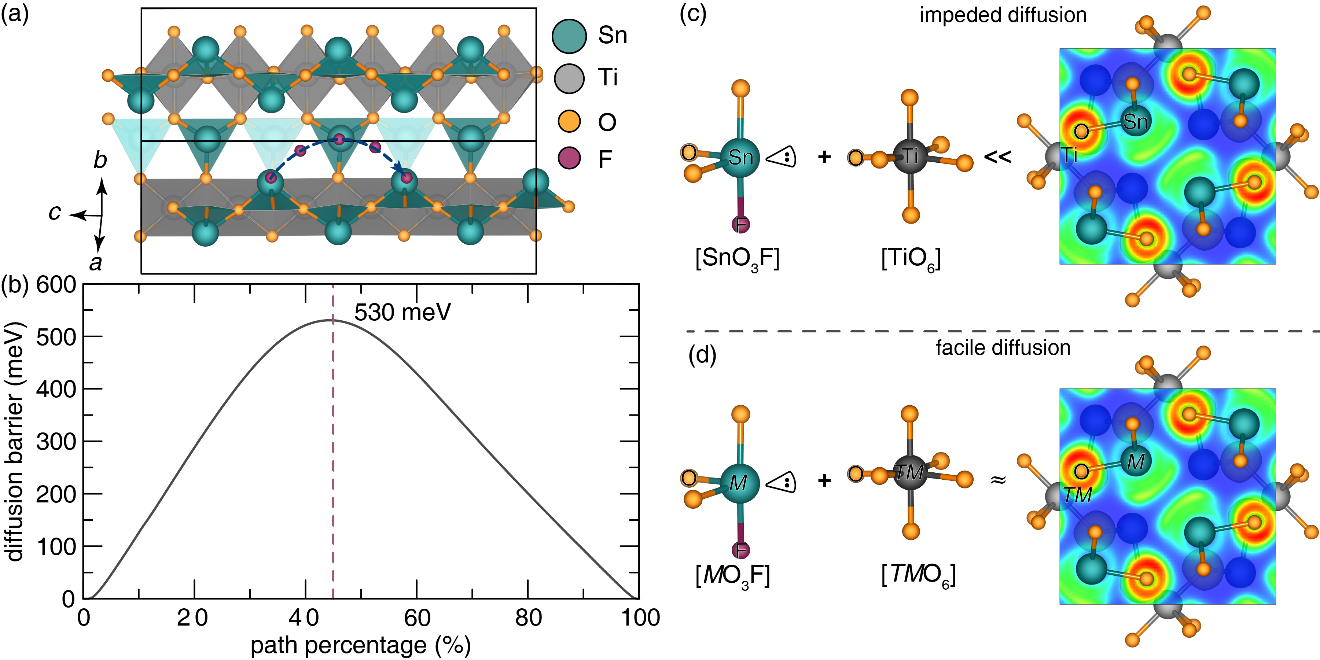 |
| --- |
| **Figure S14. (a)** The pathway (dashed blue arrow) for fluoride-ion diffusion modeled using constrained *ab inito* molecular dynamics simulations yielded an **(b)** energy diffusion barrier of 530 meV. |

**References**

(1) O'Donnell, S.; Hamilton, A.; Maggard, P. A. Fast Flux Reaction Approach for the Preparation of Sn_2_TiO_4_: Tuning Particle Sizes and Photocatalytic Properties. *Journal of The Electrochemical Society* **2019**, *166* (5), H3084. DOI: 10.1149/2.0141905jes.

(2) Whittingham, M. S.; Dines, M. B. n‐Butyllithium—An Effective, General Cathode Screening Agent. *Journal of The Electrochemical Society* **1977**, *124* (9), 1387. DOI: 10.1149/1.2133659.

(3) Zaheer, W.; Agbeworvi, G.; Perez-Beltran, S.; Andrews, J. L.; Aierken, Y.; Weiland, C.; Jaye, C.; Yu, Y.-S.; Shapiro, D. A.; Fakra, S. C.; et al. Lessons Learned from FeSb_2_O_4_ on Stereoactive Lone Pairs as a Design Principle for Anion Insertion. *Cell Rep. Phys. Sci.* **2021**, *2* (10), 100592. DOI: 10.1016/j.xcrp.2021.100592.

(4) Zaheer, W.; Andrews, J. L.; Parija, A.; Hyler, F. P.; Jaye, C.; Weiland, C.; Yu, Y.-S.; Shapiro, D. A.; Fischer, D. A.; Guo, J.; et al. Reversible Room-Temperature Fluoride-Ion Insertion in a Tunnel-Structured Transition Metal Oxide Host. *ACS Energy Letters* **2020**, *5* (8), 2520-2526. DOI: 10.1021/acsenergylett.0c01328.

(5) Agbeworvi, G.; Pakhira, A.; Hariyani, S.; Zaheer, W.; Giem, A.; Ayala, J. R.; Ponis, J. D.; Perez-Beltran, S.; Jaye, C.; Weiland, C.; et al. Stereochemical expression of Bi 6s2 lone pairs mediates fluoride-ion (De)insertion in tunnel-structured Bi2PdO4 and Bi1.6Pb0.4PtO4. *Chemical Science* **2025**, *16* (12), 5129-5141, 10.1039/D4SC08111K. DOI: 10.1039/D4SC08111K.

(6) Blakely, C. K.; Bruno, S. R.; Kraemer, S. K.; Abakumov, A. M.; Poltavets, V. V. Low-temperature solvothermal fluorination method and synthesis of La4Ni3O8Fx oxyfluorides via the La4Ni3O8 infinite-layer intermediate. *Journal of Solid State Chemistry* **2020**, *289*, 121490. DOI: <https://doi.org/10.1016/j.jssc.2020.121490>. Nowroozi, M. A.; Ivlev, S.; Rohrer, J.; Clemens, O. La_2_CoO_4_: A New Intercalation Based Cathode Material for Fluoride Ion Batteries with Improved Cycling Stability. *Journal of Materials Chemistry A* **2018**, *6* (11), 4658-4669, 10.1039/C7TA09427B. DOI: 10.1039/C7TA09427B. Nowroozi, M. A.; Wissel, K.; Rohrer, J.; Munnangi, A. R.; Clemens, O. LaSrMnO_4_: Reversible Electrochemical Intercalation of Fluoride Ions in the Context of Fluoride Ion Batteries. *Chemistry of Materials* **2017**, *29* (8), 3441-3453. DOI: 10.1021/acs.chemmater.6b05075. Wissel, K.; Heldt, J.; Groszewicz, P. B.; Dasgupta, S.; Breitzke, H.; Donzelli, M.; Waidha, A. I.; Fortes, A. D.; Rohrer, J.; Slater, P. R.; et al. Topochemical Fluorination of La_2_NiO_4+d_: Unprecedented Ordering of Oxide and Fluoride Ions in La_2_NiO_3_F_2_. *Inorganic Chemistry* **2018**, *57* (11), 6549-6560. DOI: 10.1021/acs.inorgchem.8b00661.

(7) Rodríguez-Carvajal, J. FullProf. *CEA/Saclay, France* **2001**, *1045*, 132-146.

(8) Momma, K.; Izumi, F. VESTA 3 for Three-Dimensional Visualization of Crystal, Volumetric, and Morphology Data. *Journal of Applied Crystallography* **2011**, *44*, 1272-1276. DOI: doi.org/10.1107/S0021889811038970.

(9) Siol, S.; Mann, J.; Newman, J.; Miyayama, T.; Watanabe, K.; Schmutz, P.; Cancellieri, C.; Jeurgens, L. P. Concepts for Chemical State Analysis at Constant Probing Depth by Lab‐based XPS/HAXPES Combining Soft and Hard X‐ray Sources. *Surface and Interface Analysis* **2020**, *52* (12), 802-810.

(10) Ravel, B. Quantitative EXAFS Analysis. In *X‐Ray Absorption and X‐Ray Emission Spectroscopy*, 2016; pp 281-302.

(11) Kresse, G.; Joubert, D. From ultrasoft pseudopotentials to the projector augmented-wave method. *Physical Review B* **1999**, *59* (3), 1758-1775. DOI: 10.1103/PhysRevB.59.1758.

(12) Blöchl, P. E. Projector augmented-wave method. *Physical review B* **1994**, *50* (24), 17953.

(13) Perdew, J. P.; Burke, K.; Ernzerhof, M. Generalized gradient approximation made simple. *Physical review letters* **1996**, *77* (18), 3865.

(14) Kresse, G.; Furthmüller, J. Efficiency of ab-initio Total Energy Calculations for Metals and Semiconductors using a Plane-Wave Basis Set. *Computational Materials Science* **1996**, *6* (1), 15-50. DOI: doi.org/10.1016/0927-0256(96)00008-0.

(15) Monkhorst, H. J.; Pack, J. D. Special Points for Brillouin-zone Integrations. *Physical review B* **1976**, *13* (12), 5188. DOI: 10.1103/PhysRevB.13.5188.

(16) Heyd, J.; Scuseria, G. E.; Ernzerhof, M. Hybrid Functionals based on a Screened Coulomb Potential. *J. Chem. Phys.* **2003**, *118* (18), 8207-8215. DOI: 10.1063/1.1564060.

(17) Maintz, S.; Deringer, V. L.; Tchougréeff, A. L.; Dronskowski, R. LOBSTER: A Tool to Extract Chemical Bonding from Plane‐Wave based DFT. Wiley Online Library: 2016.

(18) Woo, T. K.; Margl, P. M.; Blöchl, P. E.; Ziegler, T. A Combined Car−Parrinello QM/MM Implementation for ab Initio Molecular Dynamics Simulations of Extended Systems:  Application to Transition Metal Catalysis. *The Journal of Physical Chemistry B* **1997**, *101* (40), 7877-7880. DOI: 10.1021/jp9717296.

(19) Ryckaert, J.-P.; Ciccotti, G.; Berendsen, H. Numerical-Integration of Cartesian Equations of Motion of a System with Constraints – Molecular-Dynamics of N-Alkanes. *Journal of Computational Physics* **1977**, *23*, 327-341. DOI: 10.1016/0021-9991(77)90098-5.
